# Manipulating the pH response of 2,3-diaminopropionic acid rich peptides to mediate highly effective gene silencing with low-toxicity^☆^

**DOI:** 10.1016/j.jconrel.2013.09.033

**Published:** 2013-12-28

**Authors:** Vincenzo Abbate, Wanling Liang, Jayneil Patel, Yun Lan, Luigi Capriotti, Valentina Iacobucci, Tam T. Bui, Poulami Chaudhuri, Laila Kudsiova, Louic S. Vermeer, Patrick F.L. Chan, Xiaole Kong, Alex F. Drake, Jenny K.W. Lam, Sukhvinder S. Bansal, A. James Mason

**Affiliations:** a Institute of Pharmaceutical Science, King's College London, Franklin-Wilkins Building, 150 Stamford Street, London SE1 9NH, UK; b Department of Pharmacology & Pharmacy, Li Ka Shing Faculty of Medicine, The University of Hong Kong, 21 Sassoon Road, Hong Kong, Hong Kong

**Keywords:** pH responsive peptides, Endocytosis, siRNA delivery, Monocytes, Manipulation of pH response

## Abstract

Cationic amphipathic pH responsive peptides possess high *in vitro* and *in vivo* nucleic acid delivery capabilities and function by forming a non-covalent complex with cargo, protecting it from nucleases, facilitating uptake *via* endocytosis and responding to endosomal acidification by being released from the complex and inserting into and disordering endosomal membranes. We have designed and synthesised peptides to show how Coulombic interactions between ionizable 2,3-diaminopropionic acid (Dap) side chains can be manipulated to tune the functional pH response of the peptides to afford optimal nucleic acid transfer and have modified the hydrogen bonding capabilities of the Dap side chains in order to reduce cytotoxicity. When compared with benchmark delivery compounds, the peptides are shown to have low toxicity and are highly effective at mediating gene silencing in adherent MCF-7 and A549 cell lines, primary human umbilical vein endothelial cells and both differentiated macrophage-like and suspension monocyte-like THP-1 cells.

## Graphical abstract

**Figure:**
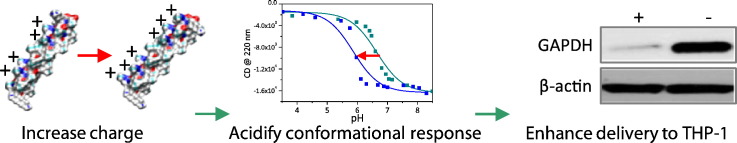

## 1 Introduction

The great therapeutic potential of RNA interference (RNAi) is threatened by waning interest from pharma in developing small interfering RNA (siRNA) based drugs [1]. The root cause of much of the frustration regarding the development of RNAi based therapies is the inability to successfully deliver siRNA to target tissues [1]. Further, gene knockdown experiments that are routinely performed in standard cell lines are often difficult to transfer to more biologically relevant model systems including primary and/or suspension human cell types.

Of the numerous non-viral delivery systems, in particular cell-penetrating peptides, conceived to overcome this hurdle [2–4], we have focussed our attention on cationic amphipathic peptides containing pH responsive residues that are capable of harnessing the changes in pH associated with endocytosis and endosomal acidification to promote release of cargo to the cell cytosol [5,6]. These peptides are capable of delivering plasmid DNA and siRNA to mammalian cell lines *in vitro* with both high efficacy and low associated toxicity [5–8], are proven to deliver antisense oligonucleotides to patient derived primary fibroblasts [9] and have also been successfully applied *in vivo* to deliver protein based vaccines adjuvanted with Toll-like receptor 9 agonist CpG oligonucleotide [10]. The observation that pH responsive peptides have such properties has led us to consider how their delivery efficacy can be improved to enable delivery to suspension cell lines and primary cells and hence widen the scope of potential *in vivo* applications. Though sharing many properties with other polycationic delivery systems, the pH responsive peptides have an additional functionality which leads to a dramatic increase in gene delivery efficacy when compared with analogous peptides comprising cationic residues, such as ornithine or lysine, which are not sensitive to pH changes in the desired range [11–13]. This behaviour is linked to pH dependent changes in conformation in solution [13], topology [14], disordering activity [11,15] in the target endosomal membrane and nucleic acid binding affinity [15,16].

To understand the role of the pH response in determining delivery efficacy, the behaviour during endosomal acidification must be considered. Acidification of the endosomes causes a dramatic change in the charge state of the carrier peptides as it switches from a nominal charge state of + 5 to either + 9 or + 11, depending on the number of pH responsive residues that have been incorporated. Since electrostatic interactions determine much of the binding affinity of the peptide for the nucleic acid, the affinity for each peptide for the cargo increases [15,16] even though the actual effect on the complex is a release of peptides [16]. Since the complex is fully saturated with peptide at neutral pH, all possible binding opportunities between nucleic acid and peptide are fulfilled. When the cationic charge on the peptide molecules doubles during endosomal acidification, only half of the bound peptides can now be accommodated by the nucleic acid as the charge state of the nucleic acid and the number of possible electrostatic interactions remain unchanged [16]. Increasing the complement of pH responsive residues should enhance peptide release from peptide–nucleic acid complexes and consequent endosomal membrane disordering and delivery. However, a further hurdle exists since the peptides may exist either as monomers or small aggregates in aqueous solutions [17]. Coulombic interactions, which exist between the protonated histidine residues when the peptide adopts an α-helix conformation in its self-associated state, constitute such a barrier that the protonation of the histidine residues and concomitant dissociation of the peptide into the monomeric and membrane accessible form may occur at a pH that is not achievable in endosomes when the histidine content is increased [15]. Increasing the histidine content also reduced the size of siRNA/peptide complexes and this, together with the more acidic response, altered the uptake route of this cargo leading to much poorer than expected delivery [8].

**Fig. 1:**
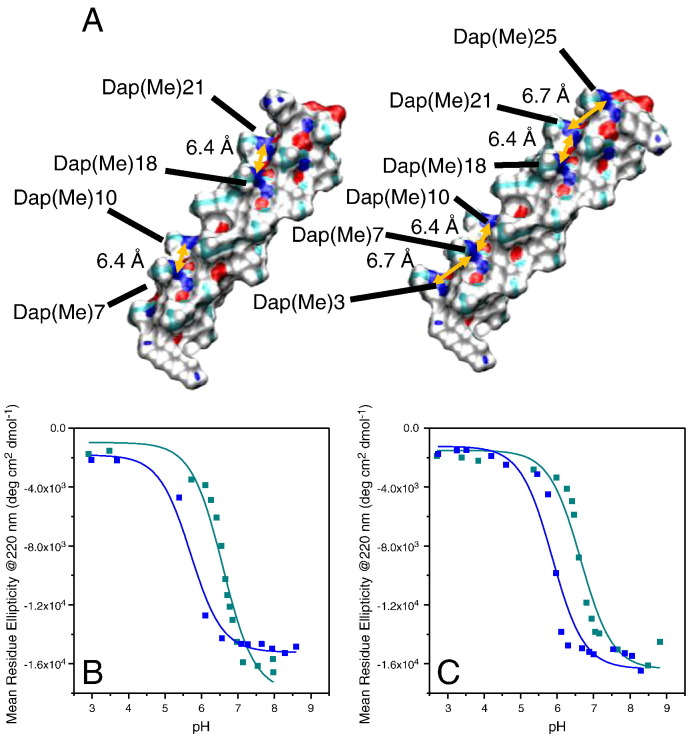
Manipulating the pH response of Dap rich peptides. A surface representation of LADap(Me)4-L1 (left) and LADap(Me)6-L1 modelled as α-helices that likely exist in an oligomeric state at neutral to basic pH in suspension (A). Coulombic interactions potentially exist between the two pairs or two triads of ionisable Dap(Me) residues located close to each other on one surface of the amphipathic α-helix and will be greater for LADap(Me)6-L1. The change in CD at 220 nm for four peptides in aqueous suspension as detected by far-UV CD spectroscopy is plotted as a function of pH; LADap4-L1 (dark cyan) and LADap6-L1 (blue) (B), LADap(Me)4-L1 (dark cyan) and LADap(Me)6-L1 (blue) (C). Increasing the complement of either the primary amine Dap (B) or the N-methylated Dap(Me) (C), from four to six residues, causes a notably more acidic conformational response.

Dap rich peptides respond at a higher pH than histidine containing analogues [13] but still respond in a range that could be exploited to drive endosomal release and this suggests a hypothesis: the same Coulombic interactions that acidified the pH response of histidine enriched peptides to a point that they were unable to act in the endosomes can be used to optimise the pH response of Dap enriched peptides (Fig. 1A). Increasing the number of Dap residues will have the added benefit that the change in nominal charge state during acidification would be increased (+ 5 to + 11 rather than + 9) and more peptides theoretically released to afford much better endosomal escape and nucleic acid delivery. We further hypothesised that the observed higher toxicity of Dap in its primary amine form [13] would be mitigated by interrupting inter-molecular hydrogen bonding networks through alkylation of Dap side chain amino groups.

Having prepared peptides to test these hypotheses, we monitored the pH dependent conformational switches of a series of Dap rich peptides with varying numbers of pH responsive elements and N-alkylation using CD spectroscopy in aqueous solution. The strongly beneficial effect of these modifications on both peptide mediated plasmid DNA and siRNA transfer is shown for a range of adherent cell lines including A549 adenocarcinomic human alveolar basal epithelial cells, MCF-7 human breast cancer cells, human umbilical vein endothelial cells (HUVECs) and differentiated THP-1 cells. Dap rich peptides with a tuned pH response are, in particular, also highly effective at mediating siRNA transfer to suspension monocyte THP-1 cells.

## 2 Material and methods

### 2.1 Materials

The peptides comprising natural amino acids (Table 1) were purchased from Pepceuticals Ltd (Nottingham, UK) as desalted grade. Fmoc-Ala-OH, Fmoc-Leu-OH, Fmoc-Lys(Boc)-OH, Boc-Lys(Boc)-OH·DCHA, NovaSyn TGR resin with a modified Rink linker and (2-(6-Chloro-1H-benzotriazole-1-yl)-1,1,3,3-tetramethylaminium hexafluorophosphate) (HCTU) Fmoc-Dap(ivDde)-OH were purchased from Merck BioSciences, NovaBiochem (Nottingham, UK) and AnaSpec (Fremont, CA). Peptide synthesis grade dimethylformamide (DMF), Oxyma, diisopropylcarbodiimide, 2,4,6-collidine and hydroxybenzotriazole were purchased from Aldrich. All other reagents were of analytical grade or better.

### 2.2 Peptide synthesis

NovaSynTGR resin with a modified Rink amide linker (0.22 mmol/g) was used for peptide synthesis on a 1 mmol scale. Fmoc deprotection was achieved with 20% piperidine in DMF (v/v) (2 × 7 min) and acylation of Fmoc protected amino acids was achieved using HCTU and collidine in the molar ratio (1:0.98:2) in peptide synthesis grade DMF using a fourfold excess for 1 h under a nitrogen atmosphere. Exceptionally, Fmoc Dap(Dde)OH was incorporated using a six fold excess or with DCI and OXYMA in a six fold excess. The acylation of Fmoc-Dap(Dde)-OH was extended for a minimum of 6 h. A Boc group was incorporated at the N-terminus to avoid undesired alkylation of the N-terminal lysine. Successful acylations and deprotections were confirmed by trinitrobenzene sulphonic acid (TNBS) test. Complete deprotection of Dde protecting groups was achieved by incubation of the resin in 2% hydrazine/DMF (v/v) (2 × 30 min) and confirmed following a small scale cleavage and analytical RP-HPLC and MALDI-TOF mass spectrometry.

### 2.3 Solid-phase N-methylation of DAP residues

Following removal of the Dde protecting group, resin bound LADap4-L1 (200 mg, 44 μmol) was treated with 2-nitrobenzene sulphonyl chloride (2-Nbs-Cl) (117 mg, 528 μmol) and collidine (106 mg, 880 μmol) in 3 ml DMF for 3 h. The resin was washed with DMF and dichloromethane (DCM) before 7-methyl-1,5,7-triazabicyclo[4.4.0]dec-5-ene (MTDB) (821 mg, 528 μmol) and methyl 4-nitrobenzene sulphonate (MSNBC) (191 mg, 880 μmol) were added in 4 ml DMF for 45 min and the resin washed again thoroughly with DMF and DCM. 20 eq. 1,8-diazabicyclo [5,4,0]undec-7-ene (DBU) (134 mg, 880 μmol) and 2-mercaptoethanol (ME) (138 mg, 1.76 mmol) were added in 4 ml DMF and a distinct yellow colour was noted. After 45 min the resin bound peptide was washed thoroughly with DMF, DCM and MeOH and dried under vacuum.

### 2.4 Synthesis of NN-dimethylated Dap containing peptides

For the synthesis of LADap(Me_2_), Fmoc-Dap(Me_2_)-OH was incorporated in the presence of HOBt/DIPCDI in a 1:1 (v/v) DMF:DMSO mixture. However, extensive recoupling was necessary to achieve complete acylation as indicated by TNBS resin tests. For the synthesis of LADap(Me_2_)6-L1 and LADap(Me_2_)6-A1 a solid-phase reductive amination approach was undertaken by adapting a previously described method [18]. Resin bound LADap6-L1 (22 μmol) was swollen in THF (0.5 ml) and treated with 37% aqueous formaldehyde (50 μl, 556 μmol) and 100 μl of a 1:1 (v/v) water:acetic acid solution. After 15 min sodium cyanoborohydride (200 μl, 1 M in THF) was added. The reaction was shaken for 3 h, and then washed with THF, water, MeOH, DCM and THF. The above procedure was repeated once more and the mixture incubated overnight. Finally the resin was washed as described above and dried *in vacuo*.

### 2.5 Cleavage and purification

The dried peptide resins were treated with TFA/phenol/H_2_O/triisopropylsilane (87.5/5/5/2.5) for 2–3 h before filtration into an excess of ice cold diethyl ether. The precipitate was centrifuged into a pellet and washed in an excess of fresh ice cold diethyl ether; this was repeated three times and finally the precipitated peptide was dissolved in 0.1% TFA and freeze-dried. Purification for both purchased and in house synthesised peptides was performed using water/acetonitrile gradients using either a Waters Symmetry™ C8, 5 μm, 7.8 × 100 mm column or a Waters SymmetryPrep™ C8, 7 μm, 19 × 300 mm column.

## 3 Results

### 3.1 Peptide design

A series of eleven cationic amphipathic peptides was used in the present study (Table 1) based upon either the most potent nucleic acid delivery peptide generated over previous studies, LAH4-L1 [11] or a shortened version, LAH [13]. Designed to adopt an amphipathic α-helix in membranes, when bound to nucleic acids and in a self-associated state at high pH in aqueous media, the histidine residues will segregate on one face and, in an idealised helical wheel representation, present an angle of 80°. Here, we have studied the effect of replacing histidine with Dap and its methylated derivatives to determine their effect on the pH response of the peptide. The three shortened peptides comprising 24 amino acids are an evolution of our previous work [13] and are designed to show the effect of N,N-di-methylation of the four Dap residues on the biophysical and gene delivery properties. To evaluate the potential for modified peptides to provide significant and substantial improvements in gene silencing capabilities, further eight peptides were conceived using the LAH4-L1 sequence as a template. Two derivatives of this peptide with four pH responsive residues were prepared; one comprising Dap (LADap4-L1) and one comprising N-methylation of Dap (LADap(Me)4-L1). Our previous work [13] suggested that peptides comprising four Dap residues only would have a pH response that is a little high to function optimally as a pH switch during endocytosis. However, further work indicates that increasing the number of pH responsive residues from four to six can cause a much more acidic pH response when the hydrophobicity of the peptide is adjusted accordingly [15]. The final series of peptides therefore is based on a LAH6-L1 peptide, which has been obtained by replacing two alanine residues with histidines, rearranging the three C-terminal residues and adjusting the hydrophobicity of the peptide by replacing two further alanines with leucines. The resulting peptide has an average hydrophobicity of 0.02 on the Eisenberg [17] scale and can be compared with LAH4-L1 at 0.05, LAH at 0.027 and is slightly less hydrophobic than the LAH6 peptides in the previous study [15]. The series comprising six pH responsive amino acids is analogous to the series described above comprising four residues with the exception that two di-methyl Dap derivatives LADap(Me_2_)6-L1 and the more hydrophilic LADap(Me_2_)6-A1 were successfully prepared.

### 3.2 Peptide synthesis

**Scheme 1:**
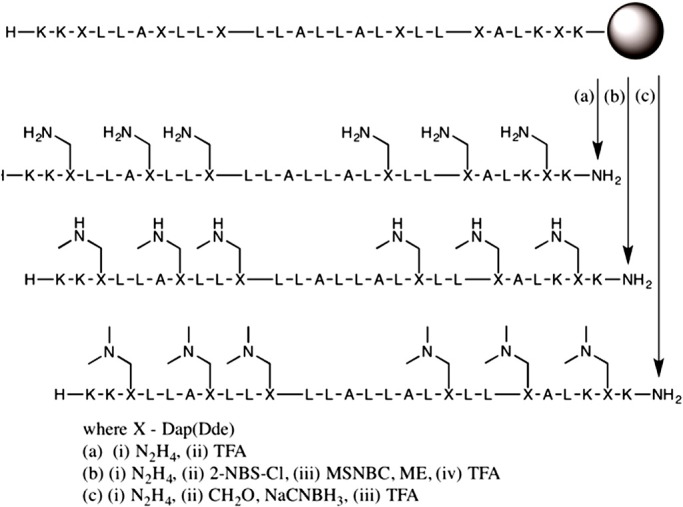
Overview of the synthetic route for obtaining three different peptides from a single assembled resin–peptide sequence. The N-terminus and lysine residues were Boc protected. Dap (X) is Dde protected and sequential treatment with three different combinations of reagent provides either non-methylated-, N-methylated- or N,N-dimethylated-Dap containing cationic amphipathic peptides.

The solid phase syntheses of short peptides containing a single monomethylated, dimethylated and trimethylated Dap have previously been reported and the synthesis of Dap has been achieved by ring opening reaction of β-lactones derived from serine [18]. Furthermore the 2-nitrobenzenesulphonyl group has been utilised for the mono alkylation of the side chain amines using both solution and solid phase [19]. Reductive amination protocols have been utilised for the synthesis of alkylated amine side chains again both in solution and on the solid phase [20–22]. However, while the amino acids derivatives are readily accessible and can be incorporated into model peptides, their incorporation into complex peptides is more challenging. Incorporation of Fmoc Dap(Boc)OH into the peptides described was straightforward; however the incorporation of Fmoc Dap(Me_2_) was problematic with incomplete acylations and required extended reaction times. A wide range of activation chemistries was investigated in order to optimise the reaction conditions. The best conditions were activation with DIC and HOBt in a mixture of DMSO and DMF. In this work, we describe a “three-birds in one-pot” solid-phase approach where we obtained three different peptides, namely non-methylated-, N-methylated- and N,N-dimethylated-Dap containing cationic amphipatic peptides from a single assembled resin–peptide sequence. The synthetic route is summarized in Scheme 1. Solid phase reactions were carried out by the synthesis of the hexa-Dap(Dde) peptide and successful removal of the Dde group to afford the hexaDap-amino peptide. Subsequently, sulphonylation with 2-nitrobenzene sulphonyl chloride followed by methylation with methyl 4-nitrobenzene sulphonate and removal of the protecting group using mercaptoethanol and base afforded the hexa-N-monomethylated-Dap containing peptide. The hexa-N,N-dimethylDAP peptide was obtained by reductive amination of the hexaDap-amino peptide with formaldehyde and sodium cyanoborahydride. The synthesised peptides were purified by preparative HPLC and characterised by MALDI-TOF MS (Supp. Fig. 1/2) with yields in the following ranges: non-methylated — 36–47%, N-methylated — 13–26%, N,N-dimethylated — 9%. To our knowledge, this is the first example of multiple and selective methylation of a complex peptide using a solid-phase approach. The orthogonal strategy could be applied to any peptide sequence and further expanded to obtain diverse methylations by employing multiple and orthogonal protecting groups.

### 3.3 NN-Dimethylation of Dap substantially reduces cytotoxicity

Though not apparent for LAH, in our previous study LADap was observed to cause some cytotoxicity during transfection [13], which we suspected could be ascribed to the four Dap primary amines. Therefore, we first investigated whether reducing hydrogen-bonding opportunities at the Dap side chain amine would improve its tolerance by cells in culture. Alkylation of the Dap primary amine may however also impact on the p*K*_a_. Predictions for the side chain p*K*_a_ obtained using Marvin View 5.1.4 for three model compounds based on 2-acetoamido-3-aminopropanamide (Supp. Fig. 1A) show that the free amine is predicted to have the lowest p*K*_a_, with the NN-dimethyl derivative having a p*K*_a_ approximately 0.6 units higher. Measurement of side chain p*K*_a_ in a peptide undergoing a conformational transition associated with a change in aggregation state is hindered by the large amounts of material required for potentiometric analyses and the unfavourable line broadening observed in NMR for self-associated peptides [13]. Instead, we have observed the cooperative, pH responsive, change of conformation of cationic amphipathic peptides in aqueous solution using far-UV CD (Supp. Fig. 1B/C). LADap (Sup. Fig. 1B) and LADap(Me_2_) (Supp. Fig. 1C) adopt an α-helix conformation when dissolved in neutral or slightly basic aqueous solution and, when titrated with acid, an increasingly disordered conformation is observed. The CD intensity at 220 nm is considered indicative of α-helix content and can be plotted as a function of pH (Supp. Fig. 1D/E). The midpoint of this conformational transition, p*K*_α_, has been observed to be closely related to the side chain p*K*_a_ when this latter information has been tractable and is an important determinant of nucleic acid transfer efficacy as it describes the transition from a self-associated form which is the trigger for effective disruption of the endosomal membrane [14,23]. Although other interactions may be expected to contribute to the p*K*_α_ when compared with the p*K*_a_, an increase of the same magnitude (~ 0.6 pH units) is seen in both the p*K*_α_ for the Dap rich peptide and the p*K*_a_ for the model compound following dimethylation (Supp. Fig. 1D/E; Table 1).

The p*K*_α_ of LADap(Me_2_) is raised by N,N-dimethylation and is likely to be too high to substantially aid endosomal release; dimethylation nevertheless has beneficial effects on the nucleic acid transfer properties of the resulting peptide. Delivery of *luciferase* reporter gene to adenocarcinomic human alveolar basal epithelial A549 cells indicates that both Dap rich peptides are effective at mediating gene transfer but, when considered in terms of luciferase activity per mg of protein, their performance is substantially inferior to that of Lipofectamine 2000™ (4:1; volume to weight DNA) and LAH4-L1 (Sup. Fig. 1F). However, the protein content of the cells, commonly used to calibrate the amount of specific luciferase activity, can be misleading. When the protein content of the wells is plotted and compared with that of untreated cells (Sup. Fig. 1G), a clearer picture emerges indicating that peptide-mediated nucleic acid transfer causes dose dependent cytotoxicity. Transfected cells would not be expected to have lower protein content than untreated cells and hence significant reductions in cell protein content are a strong indicator of cytotoxicity. LADap is notably more toxic to A549 cells when compared with the dimethylated LADap(Me_2_). Accordingly, the transfection efficacies of LADap and Lipofectamine 2000™ (4:1) in this experiment are substantially overstated. When luciferase activity is plotted per well (Supp. Fig. 1H), not only is the robust and non-toxic transfection efficacy of LAH4-L1 evident, offering a 13.0 fold improvement over Lipofectamine 2000™, but also the advantages of LADap(Me_2_) over LADap are clear. The *luciferase* delivery efficacy of LADap(Me_2_) however was insufficient for it to be considered for siRNA delivery and further modifications were conceived.

### 3.4 The pH response of Dap rich peptides can be tuned

Using LAH4-L1 as a template, we investigated the effect of replacing histidine residues with either Dap or N-methyl Dap on the cooperative, pH dependent, conformational response in solution and investigated whether this conformational response could be tuned by increasing the Dap complement in the peptide, and consequently increasing the Coulombic interactions expected between Dap or Dap(Me) residues located close to each other in space (Fig. 1A). At the same time, we mitigated the expected reduction in hydrophobicity by increasing the number of leucine residues at the expense of alanine residues. The overall aim was to obtain peptides with the appropriate hydrophobicity to afford favourable interactions with nucleic acids and membranes, switch from a nominal charge of + 5 to + 11 during endosomal acidification and have a conformational transition between pH 5 and pH 6.

Far-UV CD spectra were obtained for LAH4-L1, LAH6-L1 and the Dap and mono-methyl Dap containing analogues in aqueous Tris amine buffer at various pH (Supp. Fig. 4). The pH dependent conformational responses, reflected in the CD intensity at 220 nm, were plotted and revealed the effect of incorporating either four or six Dap or mono-methyl Dap residues in the peptide primary sequence (Fig. 1; Table 1). For LAH4-L1, the p*K*_α_ = 5.29 ± 0.25 and is in agreement with previous work while p*K*_α_ = 4.45 ± 0.25 for LAH6-L1 is substantially higher than that reported recently for LAH6 peptides of greater hydrophobicity [14]. The response of LADap4-L1 at 6.64 ± 0.08 is substantially more basic than the histidine-containing analogue. N-methylation of the Dap side chain did not alter the pH response significantly (Fig. 1B/C). Notably however the two peptides containing six Dap or six N-methyl Dap residues had a much more acidic response when compared with the analogues comprising only four such residues with midpoints for the conformational transition at pH = 5.67 ± 0.17 and 5.77 ± 0.09 respectively, a drop of ≈ 1 pH unit. Taken together this indicates that, when located close together in space, increasing the number of pH responsive residues can indeed be used to tune the p*K*_α_.

The pH response of the six N,N-dimethyl-Dap containing peptide, LADap(Me_2_)6-L1, was not determined as this peptide was found to be insoluble in aqueous media as the six N,N-dimethyl-Dap residues increased the peptide hydrophobicity dramatically. The removal of hydrogen-bonding opportunities from the Dap side chain had a substantial effect on the peptide hydrophobicity, as suggested by the large increase in the peptide retention time when analysed by HPLC (Supp. Fig. 5). A further six N,N-dimethyl-Dap variant, LADap(Me_2_)6-A1 was prepared in an attempt to circumvent this by reducing the hydrophobicity by substituting four leucines with alanine. While a small improvement in solubility was noted and the HPLC retention time reduced, this peptide remained insufficiently soluble for biophysical or biological studies. LADap(Me_2_)6-L1 was however soluble in 50% trifluoroethanol and a far-UV CD spectrum was obtained (Supp. Fig. 6) which indicated the desired α-helix conformation would theoretically be obtainable if it could be delivered to the endosomal membrane.

We have previously linked the membrane disordering capabilities of pH responsive peptide to their nucleic acid transfer capabilities [11] and that the midpoint of the pH dependent peptide induced membrane disordering p*K*_mem_ reflects the contributions of both peptide and membrane composition to the pH dependent membrane activity [15]. The incorporation of chain deuterated lipids such as POPC-d31 or POPS-d31 along with cholesterol in lipid bilayers designed to mimic the endosomal membrane allows the effect of pH dependent changes in peptide behaviour on either zwitterionic or anionic lipids to be monitored. ^2^H spin echo NMR spectra of multi lamellar vesicles are characterised by a series of quadrupolar splittings that correspond to the deuterated groups which are located at increasing depth in the membrane and which decrease with magnitude towards the hydrophobic core of the membrane where groups are more disordered. When order parameters are averaged over the whole acyl chain and plotted as a function of pH, the effect of pH responsive peptides can be observed (Supp. Fig. 7A/B). As observed previously for histidine rich peptides [15], both LADap(Me)4-L1 and LADap(Me)6-L1 effectively increase the disorder of such membranes at acidic pH with a substantially more basic response detected for both peptides in anionic membranes (Supp. Fig. 7B) compared with zwitterionic membranes (Supp. Fig. 7A). Interestingly, in contrast with histidine rich peptides (15), LADap(Me)6-L1 consistently responds at a more basic pH when compared with LADap(Me)4-L1. This may be related to the exothermic heat of binding resulting from electrostatic interactions between peptide and lipids that develop at the membrane surface during protonation that has been observed to be particularly strong for Dap [24]. Nevertheless, both peptides are expected to be capable of disordering their target membranes at pH that are readily achievable during endocytosis.

### 3.5 The tuned pH responsive peptides have improved nucleic acid transfer capabilities

**Fig. 2:**
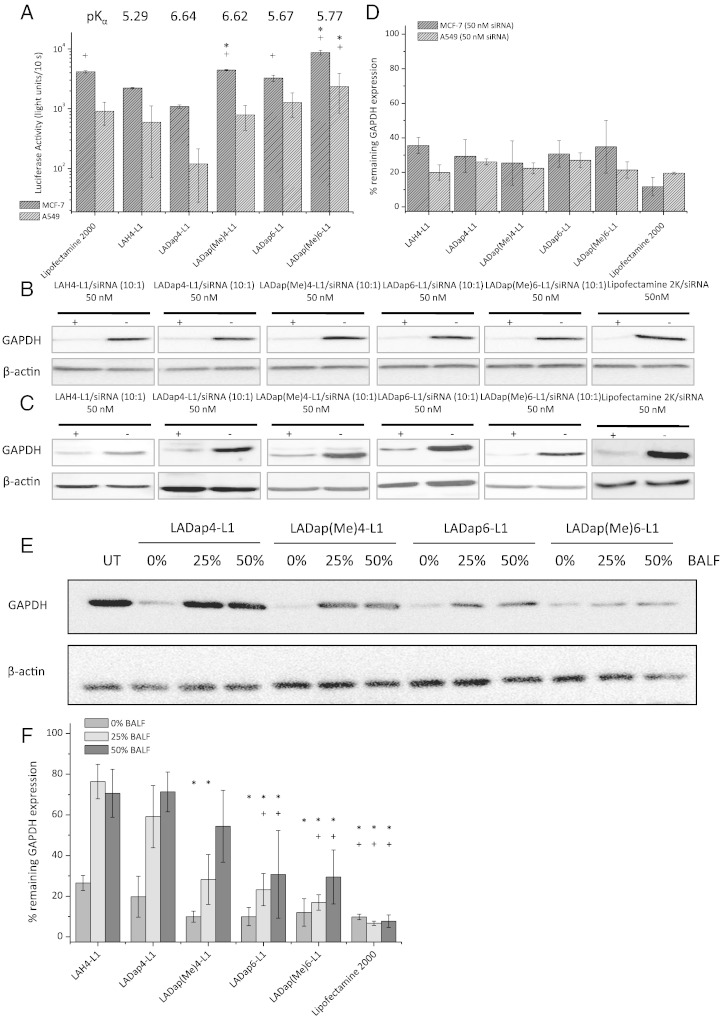
Nucleic acid transfer activity to various cell lines. Transfection of MCF-7 or A549 cells with a *luciferase* reporter gene by Lipofectamine 2000™, LAH4-L1 and a variety of Dap and N-methyl-Dap rich peptides (A). Specific knockdown of GAPDH in A549 (B) and MCF-7 (C) cells mediated by four Dap or Dap(Me) rich peptides or Lipofectamine 2000™ benchmarks and assessed by Western blot 72 h post transfection. Cells were transfected with peptide/siRNA complexes containing GAPDH siRNA (+) or negative control siRNA (−) and β-actin served as an internal control for equal protein loading and off target effects. Densitometry results are shown as an average of three independently repeated experiments (D). The effect of 0, 25 or 50% bronchoalveolar lavage fluid (BALF) on siRNA transfection of A549 cells is shown by Western blot (E) and its densitometry (F). (*) and (+) *p* < 0.05 improvement relative to LAH4-L1 and LADap4-L1 respectively.

The ability of the peptides to mediate nucleic acid transfer was first assessed by monitoring the delivery of *luciferase* reporter gene to both A549 and MCF-7 human breast cancer cells (Fig. 2A). Lipofectamine 2000™ was used here as a benchmark and at an optimised volume to weight DNA ratio of 2:1 to minimise toxicity seen in the earlier experiments (Supp. Fig. 1H). LAH4-L1 was effective at mediating luciferase expression in both cell types but was somewhat inferior to Lipofectamine 2000™ when used at the optimised ratios. Consistent with the high p*K*_α_ and the greater expected toxicity of the Dap side chain free amine, LADap4-L1 was consistently less effective than LAH4-L1 in mediating delivery to both cell types. However, N-methylation of the Dap side chain led to a substantial improvement in delivery with LADap(Me)4-L1 providing a 4.1 and 2.0 fold improvement over LADap4-L1 and LAH4-L1 respectively for MCF-7 cells (*p* < 0.05) and efficacy that matched that of LAH4-L1 for A549 cells. Increasing the Dap or N-methyl Dap content in the peptide and the concomitant acidification of the conformational response led to a further increase in peptide mediated luciferase expression with the N-methylated peptide again offering the better performance. LADap(Me)6-L1 provided an 8.0 or 19.7 fold improvement over LADap4-L1 and a 2.1 or 2.6 fold improvement over Lipofectamine 2000™ for MCF-7 or A549 cells respectively which was also significant (*p* < 0.05).

**Fig. 3:**
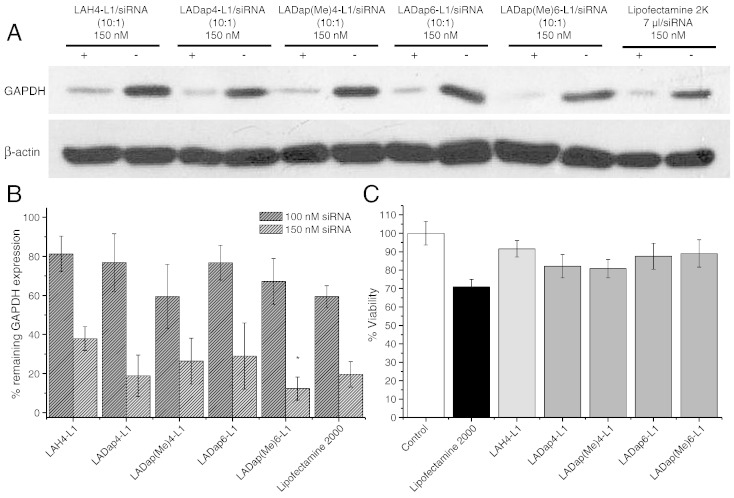
Specific knockdown of GAPDH in HUVECs mediated by four Dap or Dap(Me) rich peptides or Lipofectamine 2000™ benchmark and assessed by Western blot either 72 or 48 h post transfection for cells transfected with 100 or 150 nM siRNA respectively (A). Cells were transfected with peptide/siRNA complexes containing GAPDH siRNA (+) or negative control siRNA (−) and β-actin served as an internal control for equal protein loading and off target effects. Densitometry results are shown as an average of three independently repeated experiments (B). (*) *p* < 0.05 improvement relative to LAH4-L1. Toxicity of non viral vectors to HUVECs was assessed using the MTT assay 24 h after a 4 h incubation in serum free media (C). Vectors were prepared as for siRNA transfection experiments with 150 nM siRNA and a peptide to siRNA weight ratio of 10:1.

**Fig. 4:**
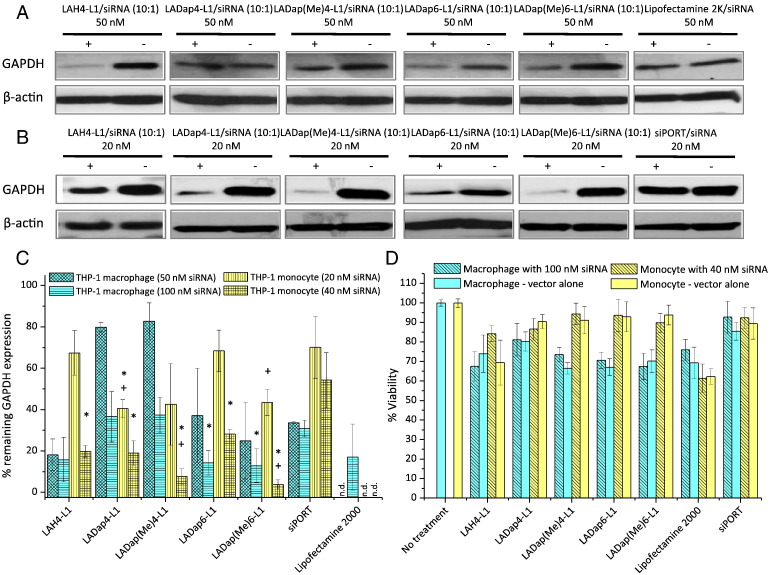
Specific knockdown of GAPDH in differentiated, adherent macrophage (A) or suspension monocyte (B) THP-1 cells mediated by four Dap or Dap(Me) rich peptides or Lipofectamine 2000™ or siPORT benchmarks and assessed by Western blot 72 h post transfection. Cells were transfected with peptide/siRNA complexes containing GAPDH siRNA (+) or negative control siRNA (−) and β-actin served as an internal control for equal protein loading and off target effects. Densitometry results are shown for as an average of three independently repeated experiments (C). (+) and (*) *p* < 0.05 improvement relative to LAH4-L1 and Lipofectamine 2000™ or siPORT benchmarks respectively. Toxicity of non viral vectors to adherent macrophage or suspension monocyte THP-1 cells was assessed using the MTT assay 24 h after a 4 h incubation (D). Vectors were prepared as for siRNA transfection experiments and the peptide to siRNA weight ratio was 10:1 in all cases.

The abilities of the peptides to successfully mediate specific silencing of GAPDH expression were then assessed in A549 cells (Fig. 2B), MCF-7 cells (Fig. 2C), HUVEC (Fig. 3A), THP-1 cells differentiated into macrophages (Fig. 4A) and undifferentiated THP-1 cells in suspension (Fig. 4B). Specific reductions in expression of GAPDH were assessed by densitometry of Western blots where the effects of administering siRNA targeting GAPDH was compared with that of non-targeting siRNA on the expression of GAPDH as endogenous reporter and β-actin as internal toxicity/specificity control (Figs. 2D, 3B, 4C). Lipofectamine 2000™ was used as a benchmark for A549 cells, MCF-7 cells, HUVECs and differentiated, adherent THP-1 cells but was unsuited for use with suspension THP-1 cells where the majority of cells were killed and insufficient protein for Western blots obtained; siPORT was used as benchmark in its place and, for comparison, also with differentiated THP-1 cells. For A549 and MCF-7 cells, all five peptides mediated effective reductions in GAPDH expression but no significant improvements over LAH4-L1 or Lipofectamine 2000™ were observed (Fig. 2B–D).

To test the suitability of the peptides for future pulmonary delivery applications *in vivo* the ability of the peptides to mediate silencing of GAPDH expression in A549 cells was repeated in the presence of bronchoalveolar lavage fluid (BALF) as a model for airway surface liquid (ASL) (Fig. 2E/F). As shown in the Western blot (Fig. 2E) and subsequent densitometry (Fig. 2F), increasing the percentage of BALF in the transfection medium caused a clear dose dependent reduction in gene silencing efficacy. This was most evident for LADap4-L1 with 50% BALF causing substantial attenuation of the GAPDH silencing. Whereas for LADap(Me)6-L1 the effect of BALF was minor and an effective reduction of GAPDH expression by 70.6% was maintained, linked to the ability of this peptide to mediate greater uptake of peptide/siRNA complexes into the cells (Supp. Fig. 13). Increasing the number of Dap or Dap(Me) residues from four to six conferred substantial and significant (*p* < 0.05) protection from the inhibitory effects of BALF although any enhanced gene silencing attributable to N-methylation of Dap under these conditions was not significant.

The ability of the peptides to mediate silencing of GAPDH expression was then tested in adherent, primary HUVECs. These hard to transfect cells required much higher siRNA concentrations with as much as 150 nM siRNA necessary for notable specific silencing to be observed (Fig. 3A). Nevertheless densitometry of the membrane (Fig. 3B) indicates the peptides performed well in comparison with the Lipofectamine 2000™ benchmark, with LADap(Me)6-L1 again mediating the most effective gene silencing and offering a significant improvement on the LAH4-L1 template peptide (*p* < 0.05). Importantly, the highly effective gene silencing afforded by the pH responsive peptides did not come at the expense of high cytotoxicity (Fig. 3C). For Lipofectamine 2000™, only 70.9 ± 4.1% cell viability was retained after treatment whereas each of the pH responsive peptides offered an improvement (*p* < 0.05) with cell viability remaining between 80.8 and 91.5%. Interestingly, while tuning the pH response, by increasing the Dap or Dap(Me) content from four to six residues, did not have a clear effect on GAPDH silencing efficacy, a possible beneficial effect on reducing cytotoxicity was detected with LADap(Me)6-L1 causing less cytotoxicity than LADap(Me)4-L1 (*p* < 0.05) and LADap6-L1 causing less than LADap4-L1 (*p* = 0.059).

The peptides were effective at mediating specific silencing of GAPDH expression in both differentiated, adherent, macrophage and suspension, monocyte THP-1 cells (Fig. 4A–C). For differentiated, adherent, macrophage THP-1 cells, effective silencing of GAPDH expression was observed only for Dap or Dap(Me) rich peptides when six of these residues were incorporated in the peptides with four residues conferring much poorer siRNA transfection capabilities (Fig. 4C). Both LADap6-L1 and LADap(Me)6-L1, along with the original LAH4-L1 template, outperformed siPORT when delivering siRNA at 100 nM but not at 50 nM, while the performance of Lipofectamine 2000™ was quite variable. For the THP-1 cells cultured in suspension as a model for monocytes, siRNA was administered at either 20 or 40 nM, in three independently repeated experiments, and a significant (*p* < 0.05) improvement over LAH4-L1 and siPORT was observed (Fig. 4C). When siRNA was administered at 20 nM, two Dap rich peptides had an improved performance while at the higher concentration all of the five peptides outperformed siPORT. Interestingly, N-methylation of the Dap sidechain caused a substantial and significant (*p* < 0.05) improvement in GAPDH silencing whether the peptides contained four or six pH responsive units; LADap(Me)6-L1 was the best performing peptide reducing GAPDH expression by 92.3 ± 3.7%.

The viability of either suspension monocyte or differentiated macrophage THP-1 cells treated with the peptide and benchmark vectors was tested using the colorimetric MTT assay in the presence and absence of siRNA cargoes (Fig. 4D). Differentiated, adherent, macrophage THP-1 cells tolerated the vectors reasonably well but significant reductions in viability (*p* < 0.05) of between 19 and 34% were observed, with the presence or absence or cargo having little effect (Fig. 4D). siPORT was much better tolerated by differentiated THP-1 cells and, when formulated with siRNA, no significant reductions in viability were observed. However this vector performed poorly as an siRNA transfection agent. In contrast, suspension, monocyte THP-1 cells were much less sensitive to the peptide vectors (Fig. 4D) and although reductions in viability were significant for all but LADap6-L1, their magnitude was substantially lower. Lipofectamine 2000™ was highly toxic to the suspension THP-1 cells, confirming the observation noted when trying to use it as a benchmark in the corresponding siRNA delivery experiments.

**Fig. 5:**
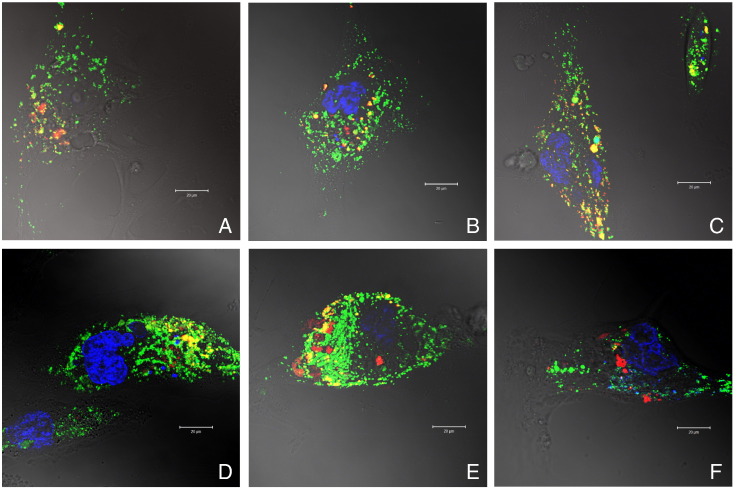
Live cell confocal imaging reveals differential localisation of LAH4-L1 or LADap(Me)6-L1 peptide/siRNA complexes (10:1 w/w) in HUVEC with reference to lysosomes. The effect of treatment with chlorpromazine and nystatin is also shown. Cy3-labelled siRNA appears red while LysoTracker® DND-26, administered 5 min prior to imaging, appears green and accumulates in cellular compartments with low internal pH. Images of HUVEC containing siRNA delivered by LAH4-L1 (A, B, C) and LADap(Me)6-L1 (D, E, F) are shown in the absence (A, D) or presence of chlorpromazine (B, E) or nystatin treatment (C, F) 24 h after transfection. Scale bar = 20 μm.

Finally the route of uptake of peptide/siRNA complexes was monitored using live cell confocal microscopy with siRNA labelled with Cy3 and acidic compartments, including mid to late endosomes as well as lysosomes, labelled with LysoTracker® DND-26 (Fig. 5). In our previous study [8] we found that differences in the distribution of siRNA clusters were more notable after 20 h post transfection. The entry of peptide/siRNA complexes to HUVECs mediated by either LAH4-L1 (Fig. 5A–C) or LADap(Me)6-L1 (Fig. 5D–F) was monitored 24 h after transfection allowing the effect of chlorpromazine (Fig. 5B/E) or nystatin (Fig. 5C/F) pre-treatment on uptake to be assessed. Both peptides mediated effective uptake of Cy3-labelled siRNA with substantial co-localisation of red siRNA with green intracellular compartments (Fig. 5A/D), particularly for LADap(Me)6-L1. Pre-treatment with chlorpromazine had little effect on delivery to HUVECs mediated by either peptide (Fig. 5B/E) in contrast with nystatin, which notably affected delivery by both peptides but in different ways. For delivery mediated by LAH4-L1, pre-treatment with nystatin caused an apparent increase in co-localisation of siRNA containing complexes with acidic compartments (Fig. 5C). Whereas, the same treatment leads to a substantial reduction in uptake, with very little co-localisation detected, when delivery was mediated by LADap(Me)6-L1 (Fig. 5F). Delivery of Cy3 labelled siRNA to differentiated macrophage THP-1 and suspension, monocyte THP-1 cells was monitored in a similar fashion (Supp. Fig. 12). For macrophage THP-1 cells, co-localisation of complexes with acidic compartments was less apparent when delivery was mediated by LADap(Me)6-L1 when compared with LAH4-L1. The method was insufficiently sensitive to detect uptake of siRNA into suspension monocyte cells.

## 4 Discussion

Since the histidine rich predecessors of the Dap rich peptides presented here have been successfully used to deliver a wide variety of cargoes, without prior conjugation, both in an *in vivo* and in an *ex vivo* study, we were interested to improve the capabilities of pH responsive peptides such that not only fibroblasts and other adherent cell lines, but also suspension cell lines and primary cells become tractable using this technology with robust gene silencing maintained in more challenging conditions. The pH responsive peptides are notable in that they are capable of not only binding non-covalently and condensing nucleic acids, preventing their degradation by endogenous nucleases and promoting cellular uptake, but are also able to promote escape from endosomes. The endosomal escape mechanism is likely to be distinct from that of the proton sponge hypothesis that has been established for other poly-cationic molecules although this may play a role [25–28]; as analogous peptides, which do not undergo pH dependent conformational changes, are much less effective at delivering nucleic acid cargo [13]. Our earlier observation, that Dap rich peptides might respond to pH changes in a range that could be exploited to drive endosomal release, suggested that enhanced delivery capabilities could be obtained if the pH response could be tuned. In practice, the Dap rich peptides behave similarly to their histidine templates and the pH response, when the number of Dap residues is increased from four to six, is acidified to the same extent as that achieved in the histidine containing analogues. In contrast with the histidine rich predecessors, the tuning of the pH response in Dap rich peptides is reflected in the improved delivery of plasmid DNA to A549 and MCF-7 cells and leads to substantial improvements in siRNA delivery either under more challenging conditions or to more challenging cell types.

While little difference between pH responsive peptides was noted in delivery of siRNA to either MCF-7 or A549 cells, substantial improvements in delivery of siRNA to the latter were observed when transfection was performed in the presence of lung surfactant containing bronchoalveolar lavage fluid (BALF) and mediated by Dap rich peptides with a tuned pH response. With pulmonary siRNA delivery an attractive route for the treatment of a wide variety of diseases affecting the airway, pH responsive peptides have been formulated as dry powders in a parallel study [29]. Transfections in the presence of BALF are designed to model the likely barrier to transfection presented by airway surface liquid (ASL). ASL covers the epithelial cells along the respiratory tract and consists mainly of phospholipids and surfactant-associated proteins which may affect the stability of siRNA/peptide complexes and hence their delivery efficacy [30,31]. Though delivery of nucleic acids to A549 cells mediated by Lipofectamine 2000™ is robust in the presence of BALF, the transfection efficiency when delivery is mediated by histidine rich peptides is weak [29]. Here we show that siRNA transfer in the presence of BALF is also weak for peptides containing only four Dap or Dap(Me) residues but when these are increased to six, good delivery efficacy is maintained. These results are in agreement with our earlier studies comparing plasmid DNA transfer [29] and highlight an advantage of tuning the pH response which is only manifested in more challenging delivery conditions. N-methylation of the Dap residues also offers a consistently observed enhancement in delivery efficacy.

The availability of cells derived from the human umbilical cord has played an important role in the development of vascular biology. HUVECs are endothelial cells that line the umbilical cord vein and have provided a critical model that has enable breakthroughs in understanding cellular and molecular events that underpin a wide variety of disease processes [32] and are considered a hard to transfect cell. They require elevated levels of siRNA for noticeable gene silencing and this leads to substantial cytotoxicity when non-viral delivery systems are used. Lipofectamine 2000™ offers robust gene silencing under the conditions used in the present study, but this is accompanied by substantial cytotoxicity. Gene silencing in HUVECs by Lipofectamine 2000™ is matched by the Dap or Dap(Me) rich peptides, with LADap(Me)6-L1 the most effective. Furthermore, the gene silencing mediated by the peptides comes with a much lower cytotoxicity cost which was itself reduced through tuning the pH response in the Dap(Me) rich peptides. HUVECs were also studied here using live cell confocal microscopy to better understand the source of the improved delivery efficacy. In our previous study we demonstrated that, in MCF-7 cells, the route of uptake for pH responsive peptide mediated siRNA delivery differs from that for DNA delivery with the former preferring clathrin independent endocytosis [8]. Furthermore, while caveolae rather than clathrin dependent endocytosis was implicated in LAH4-L1 mediated siRNA delivery, increasing the histidine content in the delivery peptide precluded entry *via* this mechanism and was suggested to be an important contributory factor in the lower than expected delivery efficacy for such peptides [8]. In the present study, chlorpromazine has little effect on uptake mediated by either LAH4-L1 or LADap(Me)6-L1, confirming that again clathrin mediated endocytosis does not provide the major route of uptake for effective delivery mediated by either peptide. Blocking caveolae dependent endocytosis of LAH4-L1/siRNA complexes did not prevent uptake but led to a much greater co-localisation with acidic compartments, most likely lysosomes. This suggests that, analogous to polyplex uptake [33], when complexes can enter *via* both clathrin and caveolae dependent endocytosis, blocking the caveolae pathway may channel complexes to the lysosomal compartments for degradation. Blocking caveolae dependent endocytosis did not trigger the same increase in co-localisation of complexes and lysosomes when delivery was mediated by LADap(Me)6-L1. This could reflect either an inability of such complexes to enter *via* clathrin dependent endocytosis, when the caveolae dependent pathway is blocked, or a much greater ability to escape from acidic compartments. The microscopy is unable to distinguish between these two possibilities but in either case, with uptake mediated by both LAH4-L1 and LADap(Me)6-L1 sensitive to blockage of caveolae dependent endocytosis, the live-cell confocal imaging study indicates that uptake mediated by peptides containing six Dap(Me) residues is likely to proceed in a manner distinct from that of analogues containing six histidine residues and suggests another important factor that contributes to their increased siRNA delivery efficacy.

The human monocytic leukaemia cell line THP-1 is widely used as a model to probe either monocyte or macrophage biology [34]. A substantial and useful improvement in siRNA delivery to macrophage THP-1 cells also accompanied the tuning of the pH response in Dap or Dap(Me) rich peptides but no improvement over the LAH4-L1 template peptide was shown.

THP-1 monocytes are of considerable interest since a wide variety of diseases may benefit from monocyte directed, siRNA based interventions. In particular, recent work has shown that silencing the chemokine receptor CCR2 in inflammatory monocytes prevents their accumulation in sites of inflammation in a mouse model [35]. The beneficial impact of this effect was demonstrated in models of atherosclerosis, coronary artery occlusion, diabetes and tumour growth [35]. Numerous other targets including, *inter alia*, Egr-1 for Alzheimer's disease [36], hepcidin for anaemia of chronic disease [37] and WEE1 for myeloid and lymphoid leukaemia [38] have been suggested following studies on THP-1 cells and effective and non-perturbing delivery agents to such cells are therefore highly sought after. While a large number of non-viral siRNA delivery systems have been developed and are widely used to mediate delivery to adherent cell lines, fewer options are available for those working with suspension cell lines where typically either lipid based transfection agents, such as Lipofectamine 2000™, or electroporation are used [34] and, while an optimised lipid nanoparticle has been successfully deployed to mediate silencing of CCR2 in an inflammatory monocyte mouse model [35], there remains a need to develop non-toxic agents that can routinely, robustly and specifically effect gene silencing in monocytes both *in vitro* and *in vivo*.

Tuning the pH response of the Dap or Dap(Me) rich peptides had less impact on delivery to monocyte THP-1 cells than that observed for macrophage THP-1. Instead, the highly efficient gene silencing mediated by LADap(Me)4-L1 and LADap(Me)6-L1 rather demonstrated the benefits of N-methylation of the Dap side chain. These two peptides are attractive candidates for further development for delivery to suspension cells since they surpassed the two benchmark delivery compounds. The relatively high cytotoxicity observed for Lipofectamine 2000™ precluded its use for siRNA delivery to monocyte THP-1 cells, with insufficient material recovered for Western blot experiments. In contrast, siPORT was much gentler but suffered from poor gene silencing efficacy.

In conclusion, using a global approach for on-resin, multiple and selective N-methylation of peptides, we prepared a family of peptides to show that overcoming the charge/self-association barrier by replacing histidine with Dap or Dap(Me) and tuning the pH dependent conformational response, by increasing the number of Dap or Dap(Me) residues, directs uptake towards the caveolae dependent endocytosis pathway and substantially improves the delivery capabilities of pH responsive peptides. N-methylation of the Dap residues notably increased gene silencing in monocyte THP-1 cells. The improvements were sufficient that the pH responsive peptides outperform the benchmark liposomal delivery agents and are able to mediate highly effective gene silencing with low associated toxicity, suggesting that they will be adaptable for *in vivo* siRNA delivery.

## Figures and Tables

**Table 1:** Sequences of LAH or LADap derivatives used in this study. pH responsive residues are marked in bold. The p*K*_α_ quoted for the peptides is the midpoint of the main conformational transition detected in solution using far-UV circular dichroism.

Peptide	Sequence	Length	p*K*_α_ at 37 °C
LAH	KKLA**H**AL**H**LLALLWL**H**LA**H**ALKKA-NH_2_	24	5.33
LADap	KKLA**X**AL**X**LLALLWL**X**LA**X**ALKKA-NH_2_	24	6.22 ± 0.16
LADap(Me_2_)	KKLA**X**AL**X**LLALLWL**X**LA**X**ALKKA-NH_2_	24	6.80 ± 0.11
LAH4-L1	KKALLA**H**AL**H**LLALLAL**H**LA**H**ALKKA-NH_2_	26	5.29 ± 0.25
LADap4-L1	KKALLA**X**AL**X**LLALLAL**X**LA**X**ALKKA-NH_2_	26	6.64 ± 0.08
LADap(Me)4-L1	KKALLA**X**AL**X**LLALLAL**X**LA**X**ALKKA-NH_2_	26	6.62 ± 0.04
LAH6-L1	KK**H**LLA**H**LL**H**LLALLAL**H**LL**H**ALK**H**K-NH_2_	26	4.45 ± 0.25
LADap6-L1	KK**X**LLA**X**LL**X**LLALLAL**X**LL**X**ALK**X**K-NH_2_	26	5.67 ± 0.17
LADap(Me)6-L1	KK**X**LLA**X**LL**X**LLALLAL**X**LL**X**ALK**X**K-NH_2_	26	5.77 ± 0.09
LADap(Me_2_)6-L1	KK**X**LLA**X**LL**X**LLALLAL**X**LL**X**ALK**X**K-NH_2_	26	n.d.
LADap(Me_2_)6-A1	KK**X**LLA**X**AL**X**ALLALLA**X**LA**X**ALK**X**K-NH_2_	26	n.d.
