# Supplementary material for: Manipulating the pH response of 2,3-diaminopropionic acid rich peptides to mediate highly effective gene silencing with low-toxicity
Source: J Control Release. 2013 Dec 28;172(3):929–38. doi: 10.1016/j.jconrel.2013.09.033 (PMC3858832; doi:10.1016/j.jconrel.2013.09.033)
Supplement: Supplementary file 1 — Supplementary figures. [file mmc1.pdf]

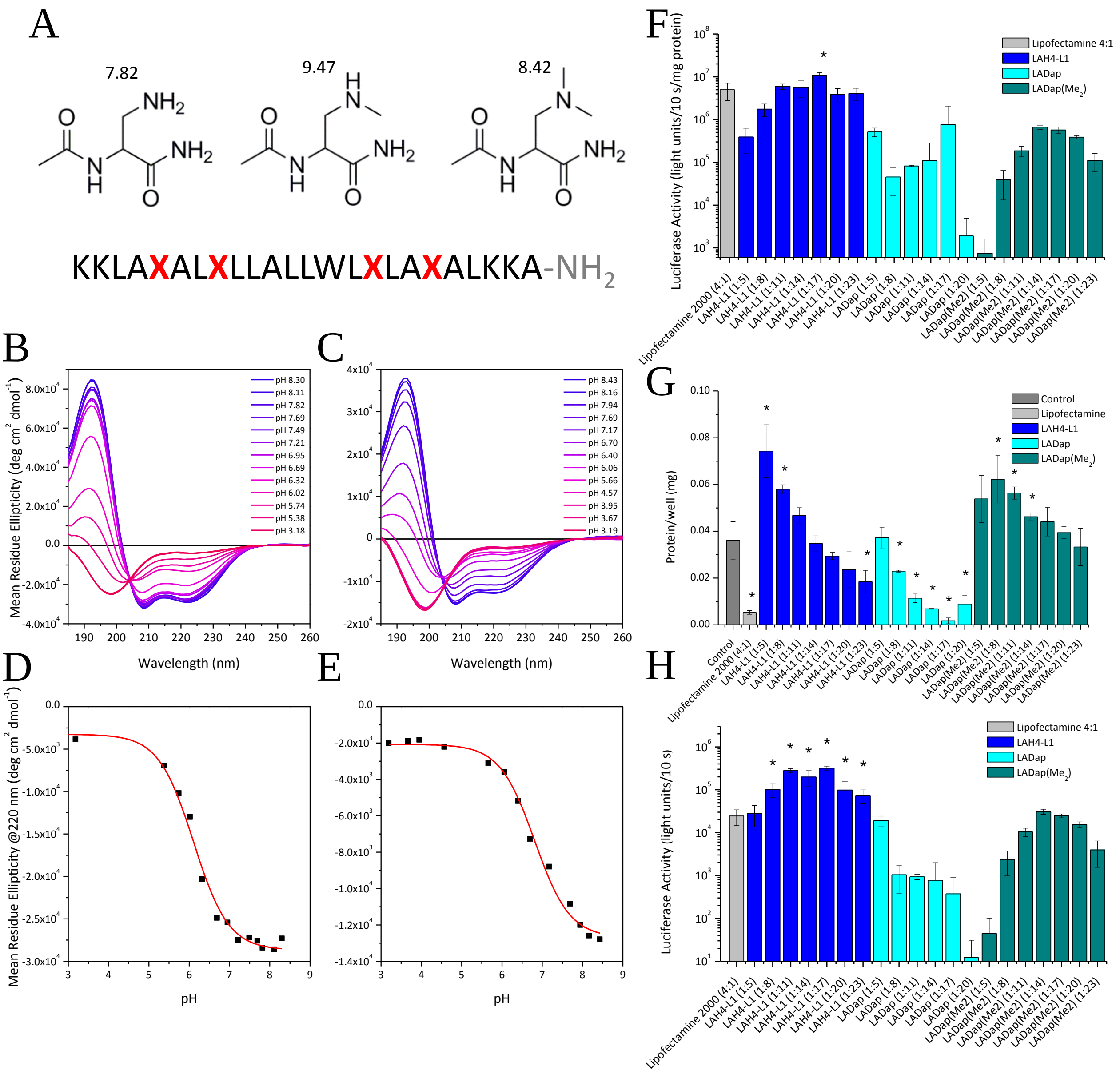

Supplementary Figure 1. *The effect of N,N-dimethylation on the predicted pK_α, conformational response and transfection of A549 cells by Dap rich peptides. The predicted side chain pK_α for 2-acetoamido-3-aminopropanamide and its N-methyl and N,N-dimethyl derivatives (**A**). When either four Dap (**B**) or N,N-dimethyl-Dap (**C**) residues are included in the peptide sequence in the positions marked with a red "X" the peptide assumes a pH dependent conformation. The midpoint of the conformational transition is determined as 6.22 ± 0.16 for LADap4 (**D**) and 6.80 ± 0.11 for LADap(Me$_2$)4 (**E**). Luciferase activity is shown as is common (**F**) as light units per mg protein. However this can mask the effect of toxicity. The protein content of the cells (**G**) is used as a measure of cytotoxicity. A reduction of protein found in a well, relative to untreated cells, provides a strong indication of cytotoxicity. * $p < 0.05$ increase (and/or decrease for protein) relative to control cells. Absolute light units (per well) may therefore be a better measure of efficacy (**H**). Lipofectamine and LADap4 are toxic to A549 cells but the N,N-dimethylation of Dap (LADap(Me$_2$)4) reduces toxicity and increases gene transfer efficacy despite the high pK_α determined in solution.*

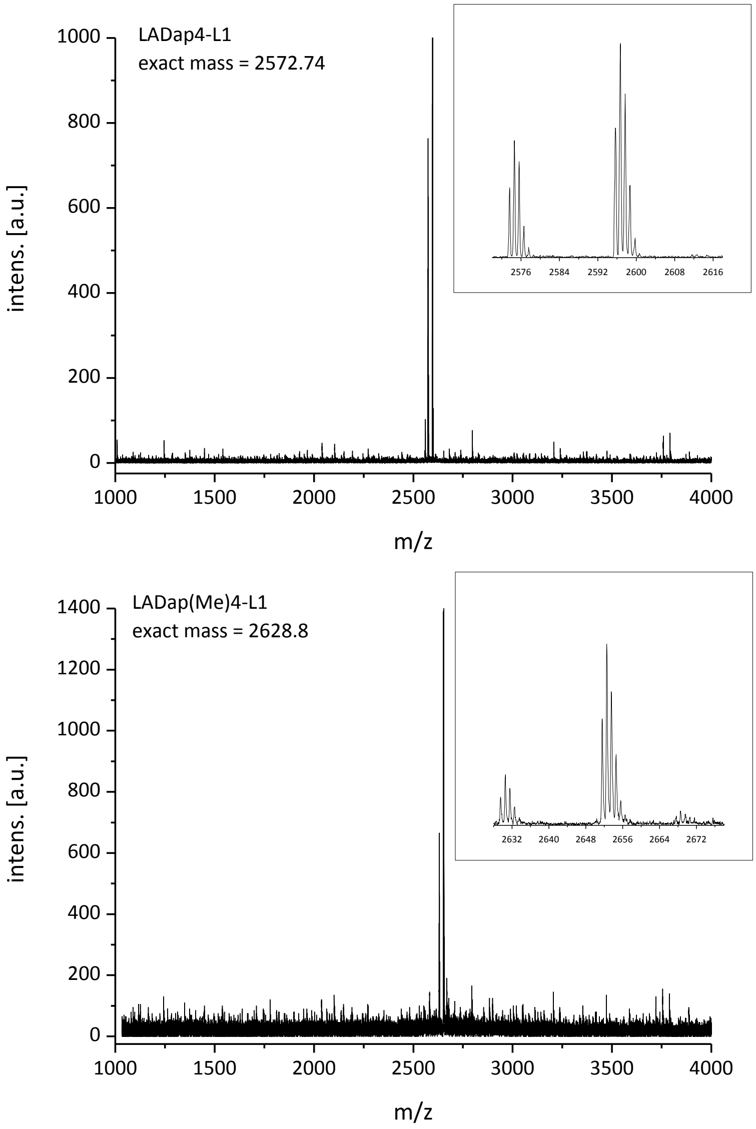

Supplementary Figure 2. *MALDI-TOF spectra of purified LADap4-L1 and LADap(Me)4-L1 peptides prepared from the same resin. The calculated exact masses are given while commonly sodium and potassium salt adducts are observed in addition to the peptide ion.*

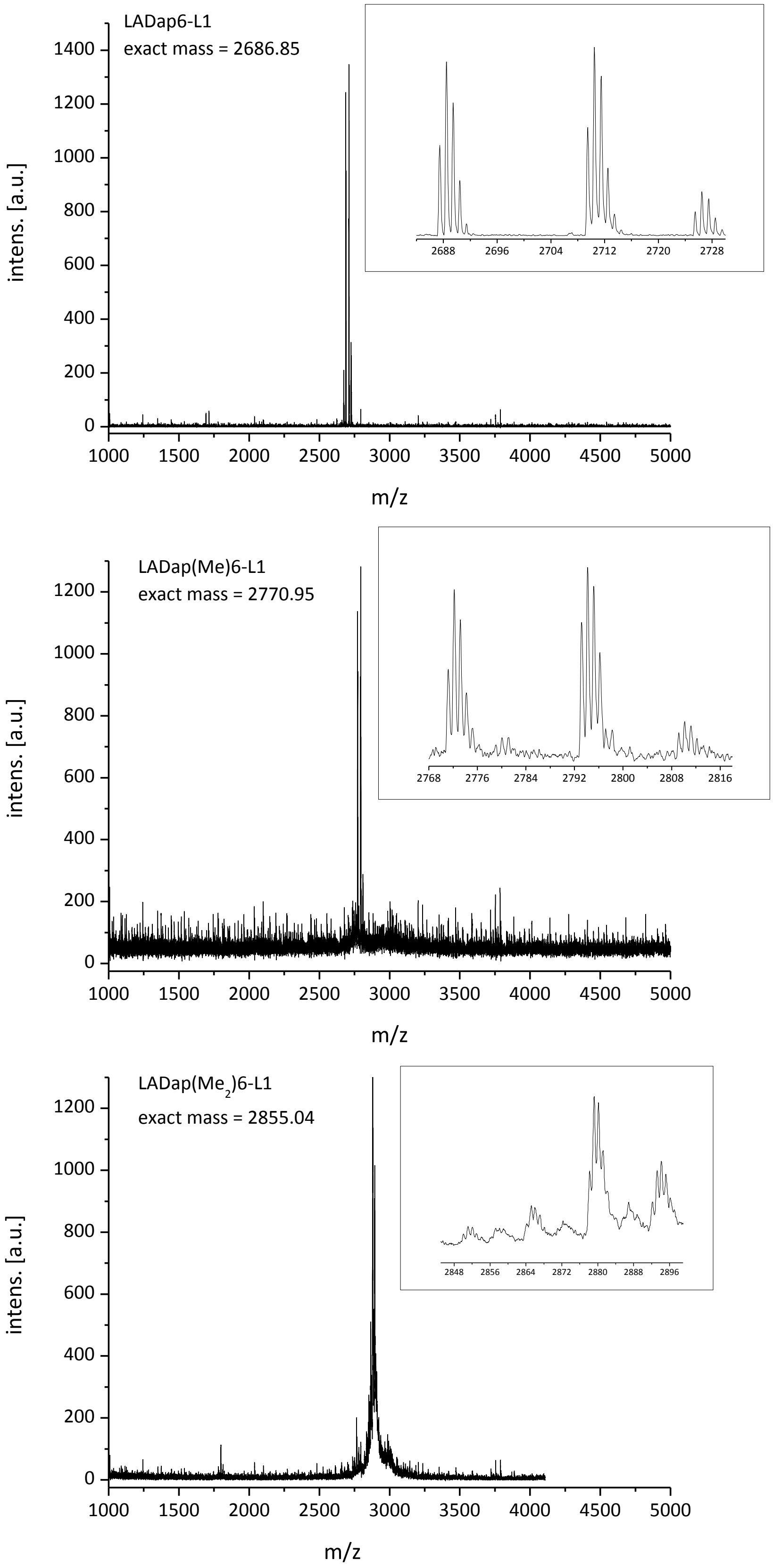

Supplementary Figure 3. *MALDI-TOF spectra of purified LADap6-L1, LADap(Me)6-L1 and LADap(Me_2)-L1 peptides prepared from the same resin. The calculated exact masses are given while commonly sodium and potassium salt adducts are observed in addition to the peptide ion. For the LADap(Me_2)6-L1 peptide, the majority of the peptide is detected as the sodium salt adduct while some small impurity, where only 5 out of 6 Dap residues are dimethylated is also detectable.*

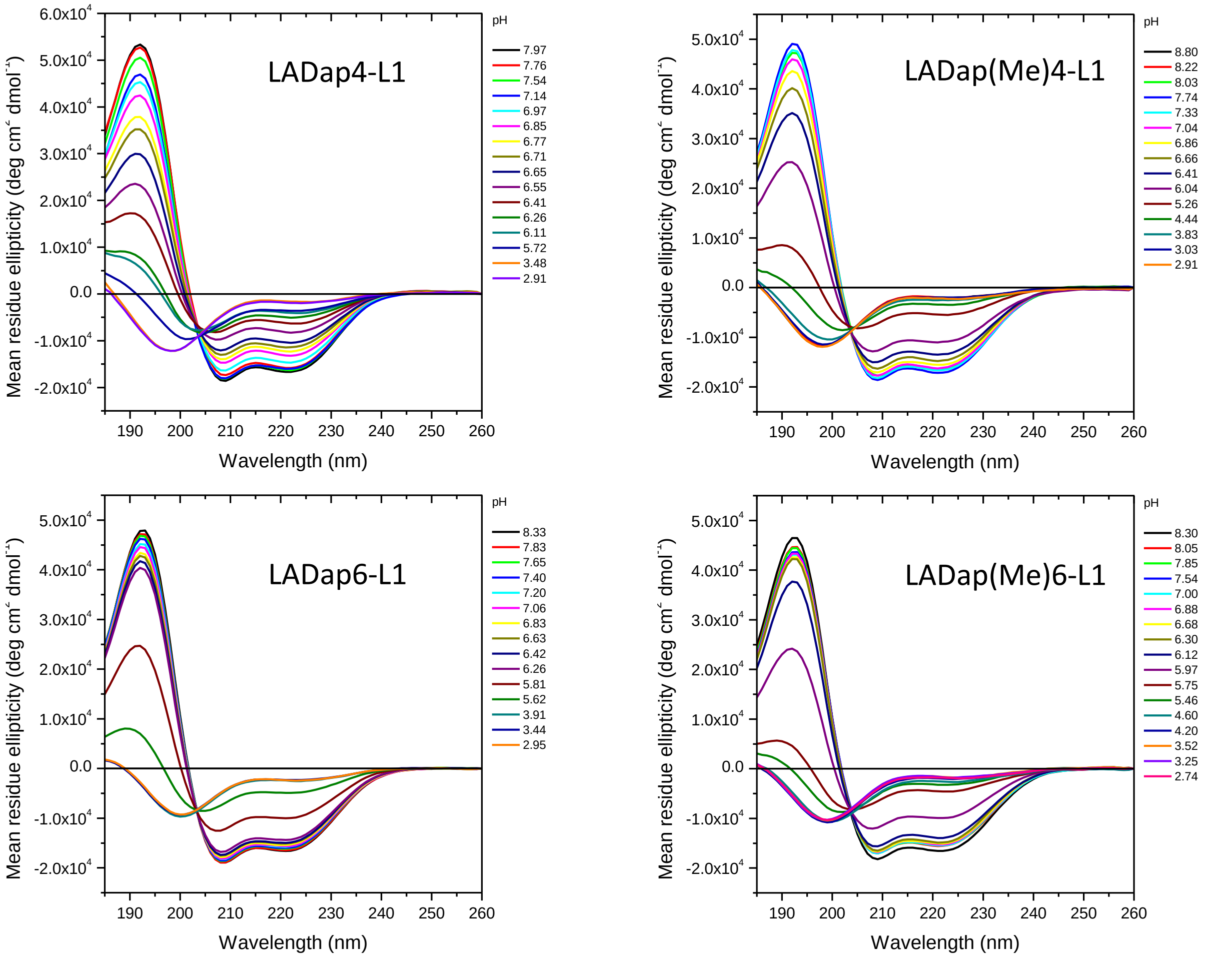

Supplementary Figure 4. *Far-UV CD spectra of 4 Dap or Dap(Me) rich pH responsive peptides in aqueous 5 mM Tris amine buffer at various pH. Spectra were recorded at 37°C. Titrations shown are representative of three or four independently repeated experiments.*

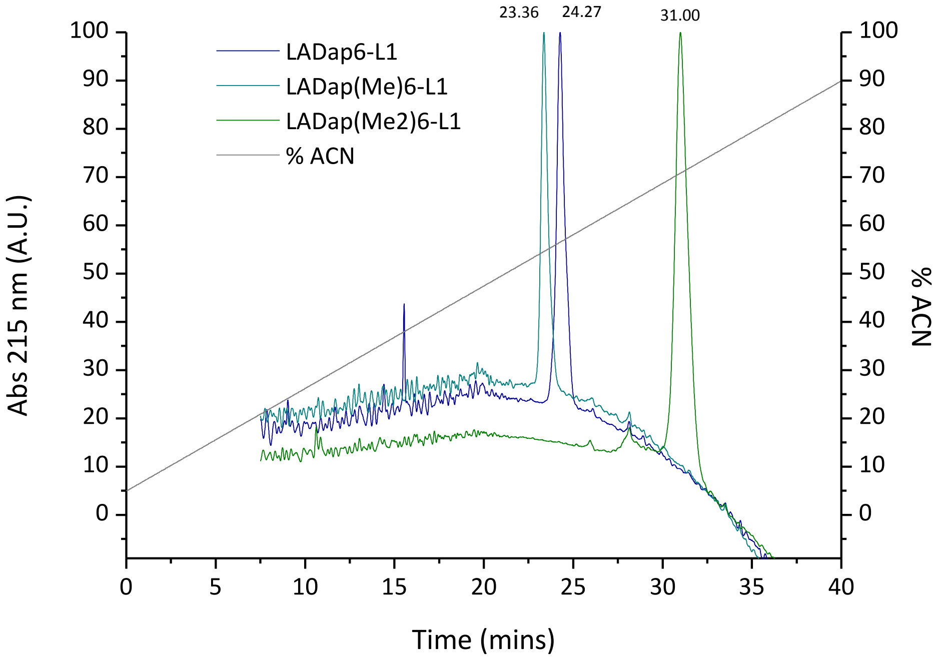

Supplementary Figure 5. *Analytical HPLC of three Dap rich peptides with differing levels of alkylation. A 5-90% water/acetonitrile gradient system was used using a Waters Symmetry™ C8, 5 µm, 7.8 x 100 mm column. The N-methylation of LADap-6-L1 to LADap(Me)6-L1 reduces the retention time of the peptide marginally however N,N-dimethylation to LADap(Me$_2$)6-L1 causes a substantial shift to longer retention time and is consistent with the insolubility of this latter peptides in aqueous media. A fourth peptide, LADap(Me$_2$)6-A1 (not shown), eluted at 28.37 minutes which demonstrated that substituting leucine with alanine residues did improve hydrophobicity but not sufficiently to make the peptide readily soluble in aqueous media.*

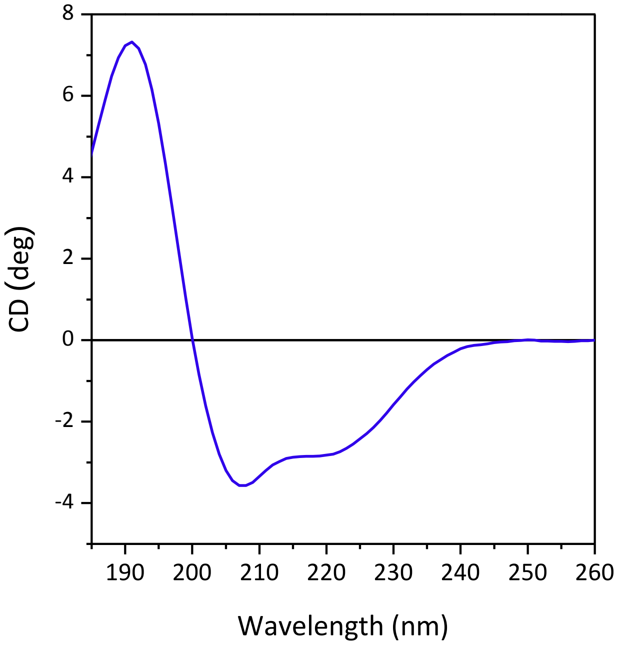

Supplementary Figure 6. *far-UV CD spectrum of LADap(Me$_2$)6-L1 in 50% TFE.*

A further six N,N-dimethyl-Dap variant, LADap(Me$_2$)6-A1 was prepared in an attempt to circumvent this by reducing the hydrophobicity by substituting four leucines with alanine. While a small improvement in solubility was noted and the HPLC retention time reduced, this peptide remained insufficiently soluble for biophysical studies. LADap(Me$_2$)6-L1 was however soluble in 50% trifluoroethanol and a far-UV CD spectrum was obtained (**Supp. Fig. 6**) which indicated the desired α-helix conformation would theoretically be obtainable if it could be delivered to the endosomal membrane.

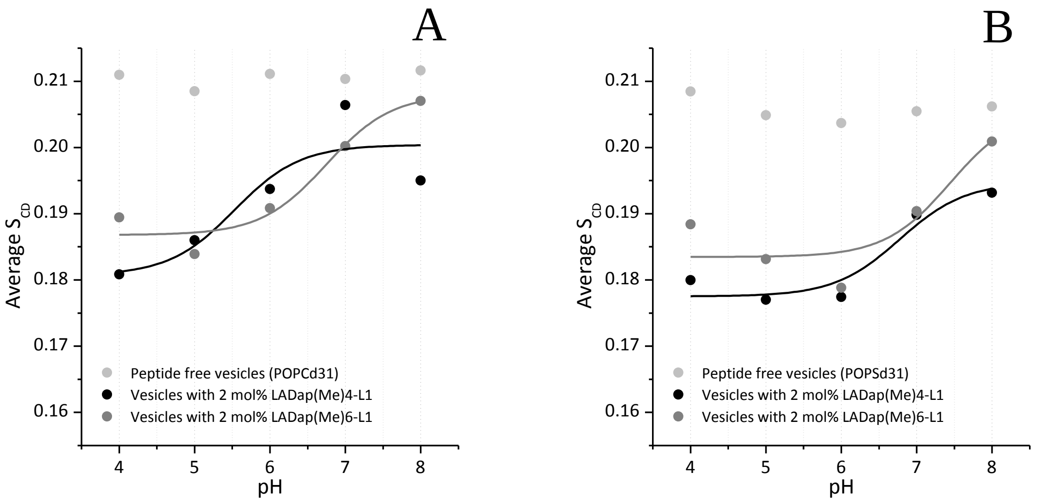

Peptide	pK_{mem} at 37° C (POPC-d31)	pK_{mem} at 37° C (POPS-d31)
LADap(Me)4-L1	5.53 ± 0.78	6.57 ± 0.16
LADap(Me)6-L1	6.75 ± 0.36	7.34 ± 0.47

Supplementary Figure 7. *Manipulating the pH response of Dap rich peptides in model membranes. Mean lipid acyl chain order parameters, obtained from solid-state ^{2}H NMR of chain deuterated lipids as reporters, are plotted as a function of pH for zwitterionic membranes comprising POPC/POPCd31/cholesterol (70:15:15) (**A**) or anionic membranes comprising POPC/POPSd31/cholesterol (70:15:15) (**B**). The pK_{mem} quoted for the peptides is the midpoint of the peptide induced disordering of the deuterated lipid. Both LADap(Me)4-L1 and LADap(Me)6-L1 undergo a pH dependent switch and induce substantial disorder at acidic pH. The presence of anionic lipids promotes a more basic pH switch in the membrane.*

The ability of LADap4-L1, LADap(Me)4-L1, LADap6-L1 and LADap(Me)6-L1 to bind and release either plasmid DNA or siRNA and protect siRNA from nucleases was tested by means of an agarose shift assay (**Supp. Fig. 8-10**). As with their histidine rich predecessors [8], the peptides shared similar DNA and siRNA binding capabilities and were able to protect siRNA effectively from the action of ribonuclease A. Interestingly, some difference in the ability of SDS to induce nucleic acid release was noted with siRNA readily released by all peptides but DNA release is often incomplete at high peptide to DNA weight ratios.

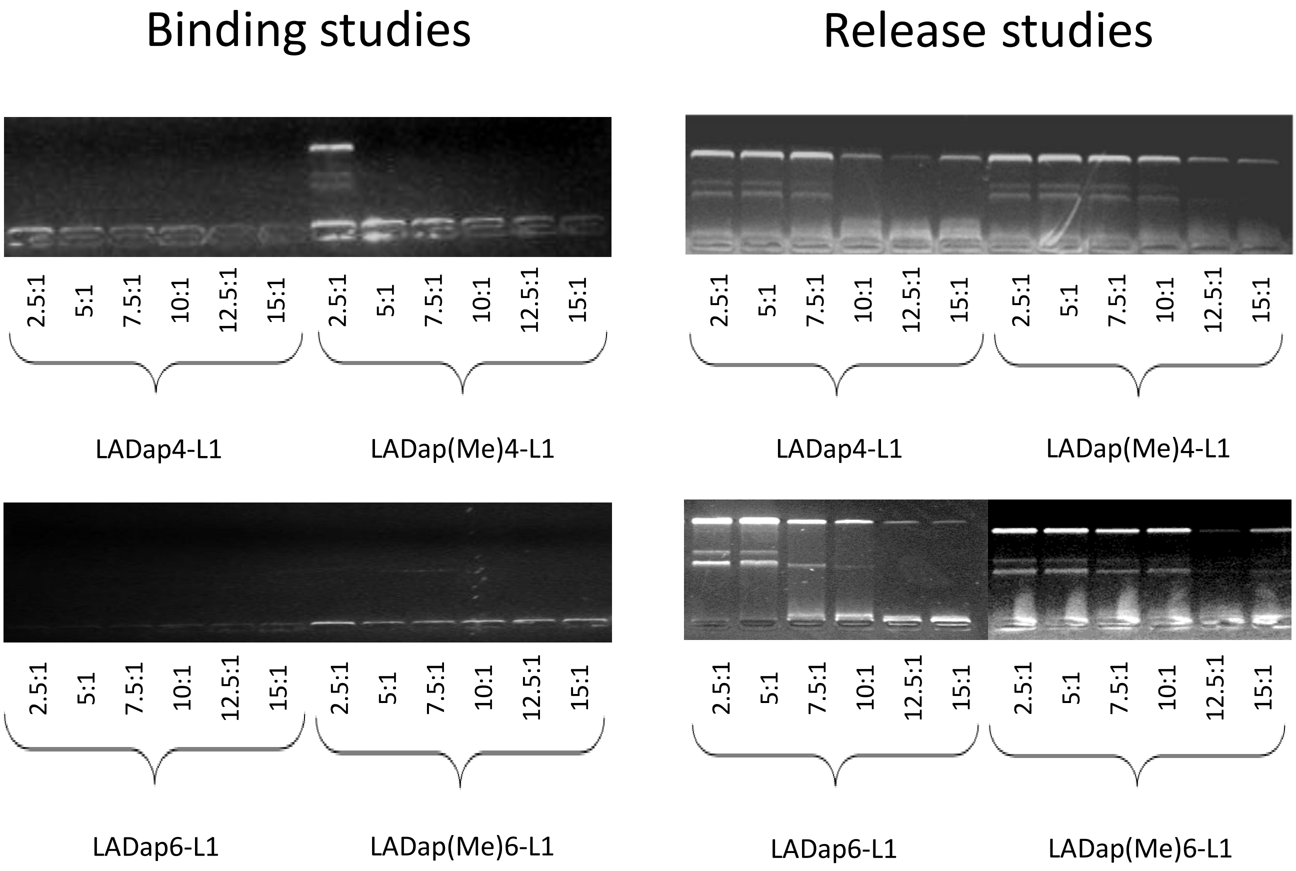

Supplementary Figure 8. *Agarose gel retardation assay of peptide/DNA binding (left panels) and release (right panels) with 5 µl 2 mM SDS. Peptide/DNA complexes were prepared at different weight/weight ratios. Electrophoresis was carried (running south to north on the figures) out at 100 V for 20 min and the gel stained with GelRed™. All four peptides effectively bind and retard the migration of DNA in the agarose gel . At lower peptide to nucleic acid weight ratios, SDS is effective at promoting DNA release from all peptides but is lees effective at higher peptide to nucleic acid weight ratios.*

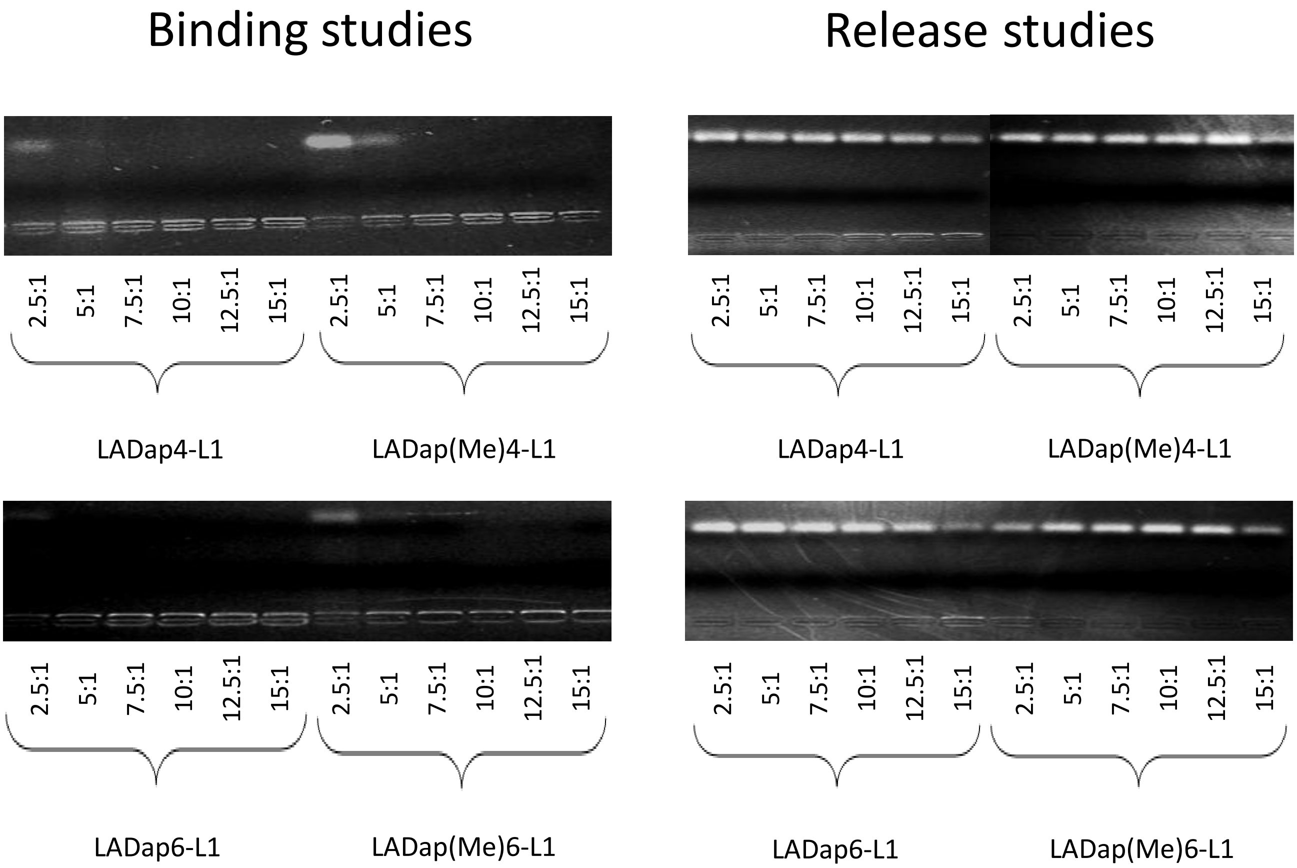

Supplementary Figure 9. *Agarose gel retardation assay of peptide/siRNA binding (left panels) and release (right panels) with 5 µl 2 mM SDS. Peptide/siRNA complexes were prepared at different weight/weight ratios. Electrophoresis was carried (running south to north on the figures) out at 100 V for 20 min and the gel stained with GelRed™. All four peptides effectively bind and retard the migration of siRNA in the agarose gel. SDS is effective at promoting siRNA release from all peptides irrespective of the peptide to nucleic acid weight ratios.*

Binding – enzymatic degradation and release studies

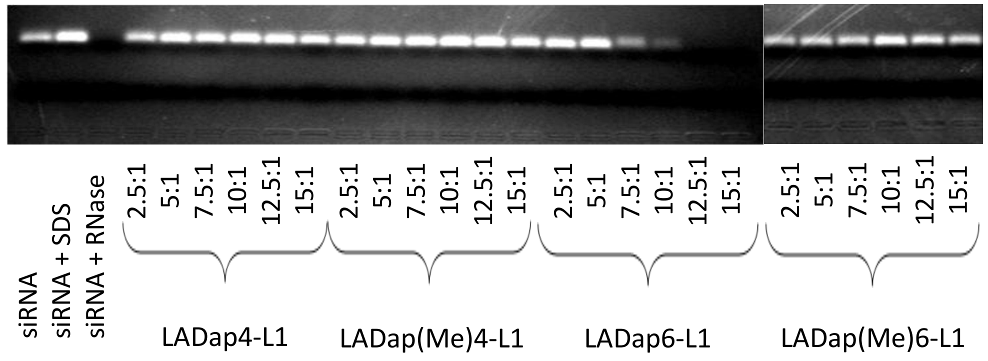

Supplementary Figure 10. *Agarose gel enzymatic degradation assay of peptide/siRNA binding and release. Peptide/siRNA complexes were prepared at different weight/weight ratios and incubated with ribonuclease A at 37°C for 30 min. The enzyme activity was stopped by ribonuclease inhibitor and the complexes dissociated using 2 mM SDS. Untreated siRNA, siRNA treated with nuclease and siRNA treated with SDS served as control. Electrophoresis was carried out (running south to north on the figures) at 100 V for 20 min and the gel stained with GelRed™. All four peptides effectively bind and protect the siRNA.*

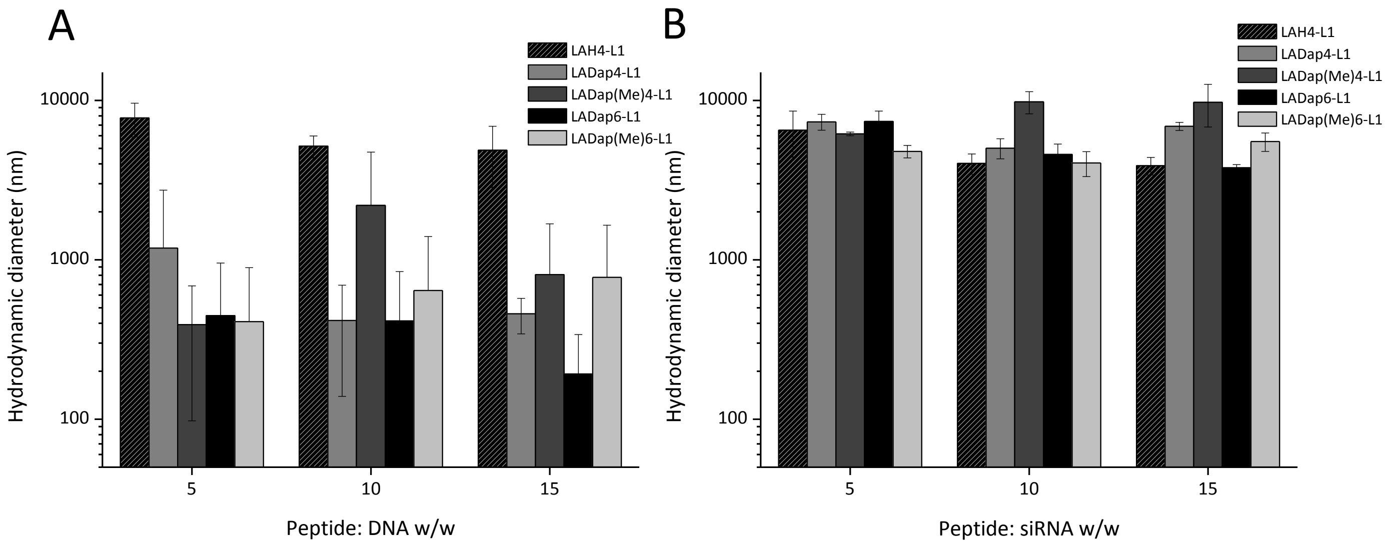

Supplementary Figure 11. *Hydrodynamic diameter of peptide/DNA (n=3) (**A**) or peptide/siRNA (n=3) (**B**) complexes prepared in 150 mM NaCl measured by photon correlation spectroscopy.*

The size of peptide/nucleic acid complexes comprising either siRNA or DNA and each of five peptides were determined at peptide to nucleic acid ratios related to the in vitro transfection experiments **(Supp. Fig. 11**). Complexes were prepared in 150 mM NaCl as a simple model for the Earle's Balanced Salt Solution on which most cell culture media are based. The complexes are cationic at all peptide to nucleic acid weight ratios with the lowest weight ratio (5:1) corresponding to a charge ratio of between 2.6 and 2.9 and the greatest weight ratio (20:1) corresponding to between 7.8 and 8.7. Hence the complexes vary between being weakly or very strongly cationic. Complexes of Dap or Dap(Me) rich peptides with plasmid DNA were, in general, around ten times smaller than those containing LAH4-L1 as the peptide component.

In contrast, when prepared in 150 mM NaCl, the siRNA/peptide complexes comprising either the histidine or Dap or Dap(Me) rich peptides were all in the same approximate size range of between around 4,000 and 10,000 nm and hence the Dap and Dap(Me) rich peptides from much larger complexes with siRNA than with plasmid DNA. LAH4-L1 siRNA complexes enter MCF-7 cells most likely through caveolae mediated endocytosis and the similar particle sizes observed for all five peptides, when complexing siRNA, suggests the Dap and Dap(Me) rich peptides will enter cells via the same or similar pathways to LAH4-L1.

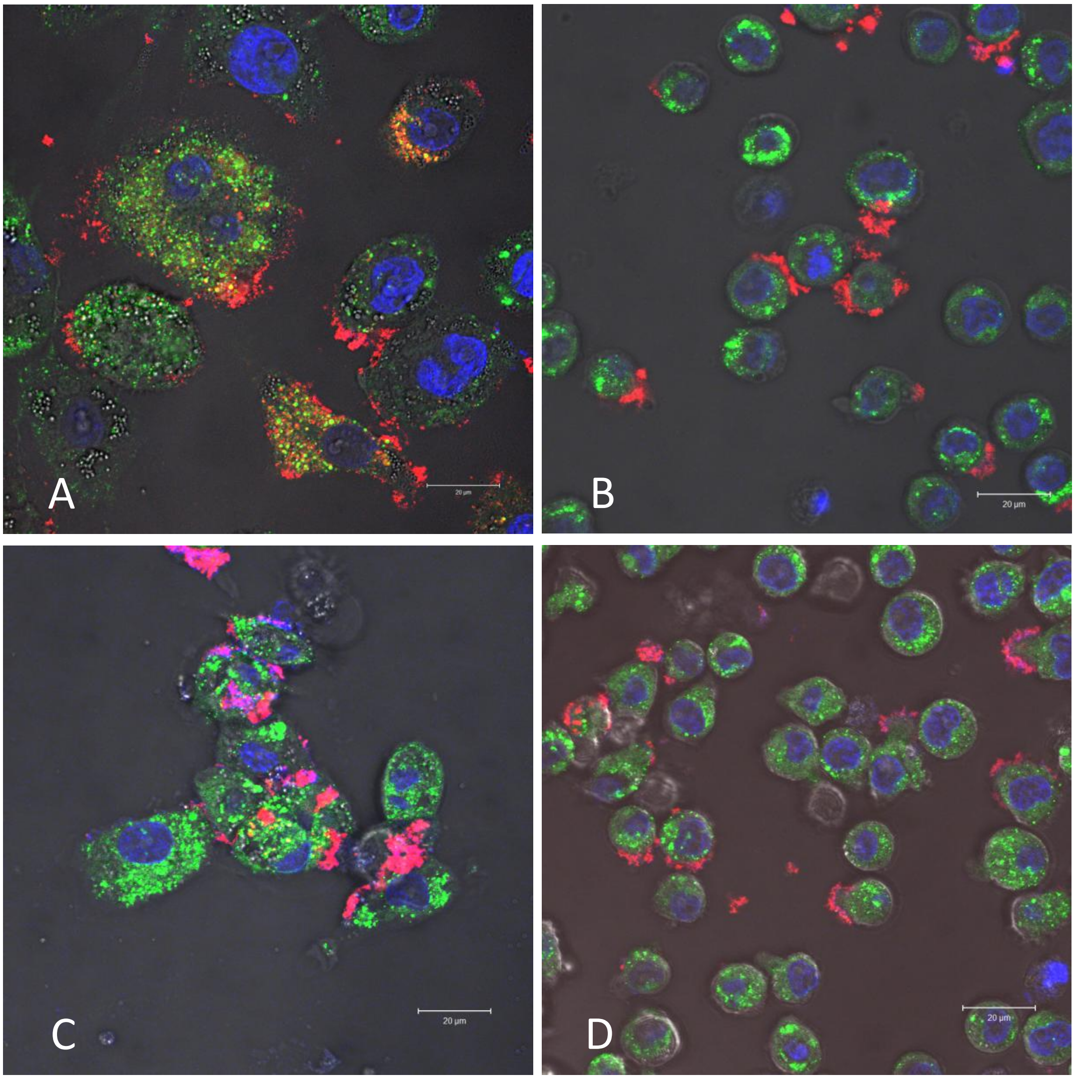

Supplementary Figure 12. *Live cell confocal images reveals localisation of LAH4-L1 or LADap(Me)6-L1 peptide/siRNA complexes (10:1 w/w) in THP-1 cells with reference to lysosomes. Cy3-labelled siRNA appears red while LysoTracker® DND-26, administered 5 minutes prior to imaging, appears green and accumulates in cellular compartments with low internal pH. Images of THP-1 containing siRNA delivered by LAH4-L1 (A, B) and LADap(Me)6-L1 (C, D) are shown for differentiated, macrophage like THP-1 (A,C) or suspension, monocyte like THP-1 (B, D) 24 hours after transfection. Scale bar = 20 µm.*

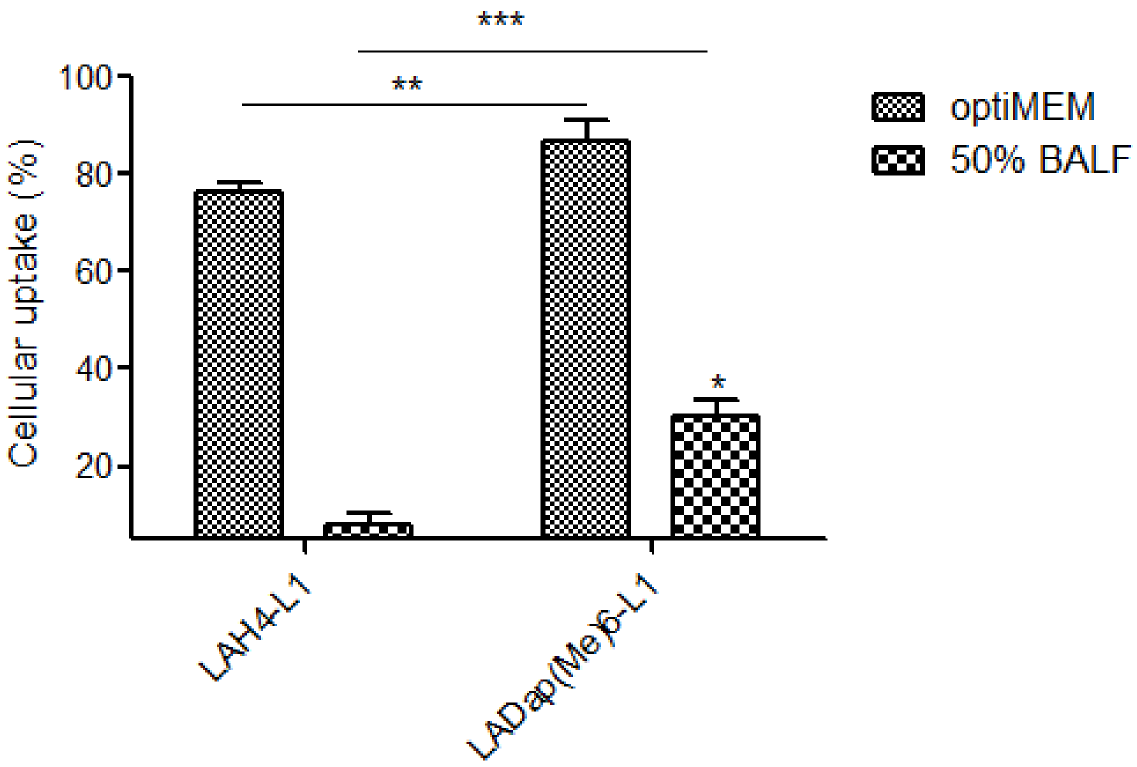

Supplementary Figure 13. *The effects of BALF on parameters related to siRNA delivery to A549 cells. Flow cytometry study* ***(A)*** *of cellular uptake of peptides/siGLO cyclophilin B siRNA complexes in opti-MEM I medium or 50% BALF (n=3). (**) p<0.01, (***) p<0.001. 100% cellular uptake was defined as that achieved with Lipofectamine 2000™.*

Bronchoalveolar lavage fluid (BALF) is used as a model for airway surface liquid [29] and causes reduction in siRNA transfection efficiency for all peptides tested in the present study. This reduction is best resisted by LADap(Me)6-L1 where silencing by 71.6 ± 13.3 % is achieved in the presence of 50% BALF in comparison with 88.0 ± 6.8% in Opti-MEM I. This can be compared with the LAH4-L1 peptide, the predecessor of the peptides presented here. LAH4-L1 mediates silencing by 73.5 ± 3.7% in Opti-MEM I but in 50% BALF, silencing is only 29.4 ± 11.8% and hence is substantially reduced.

Some explanation of the different effect of BALF comes from studying the uptake of complexes via flow cytometry of siGLO lcyclophilin B labelled siRNA complexes **(Supp. Fig. 13)** and the ability of BALF to cause release of siRNA from peptide/siRNA complexes **(Supp. Fig. 14**). A much larger proportion of peptide/siRNA complexes are taken up by A549 cells in the presence of 50% BALF when LADap(Me)6-L1 is the carrier peptide with very few LAH4-L1/siRNA complexes entering the cells. Much less of a difference is seen between these two peptides when considering the release of siRNA caused by BALF and this property does not appear to vary or determine the overall silencing efficacy and hence the electrophoretic method may substantially overestimate the true dissociation of the complexes that occurs during the transfection experiment. Nevertheless, the imperfect release of siRNA from LADap4-L1 complexes in the presence of BALF indicates this parameter may provide some future scope for reducing sensitivity to BALF.

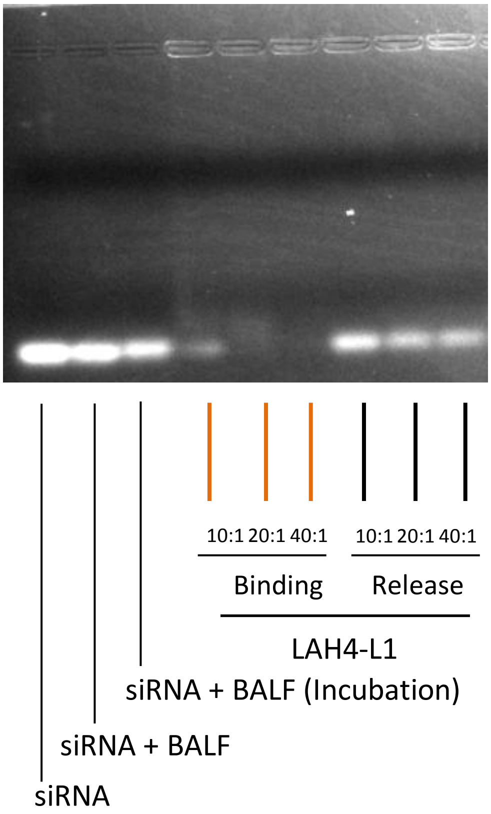

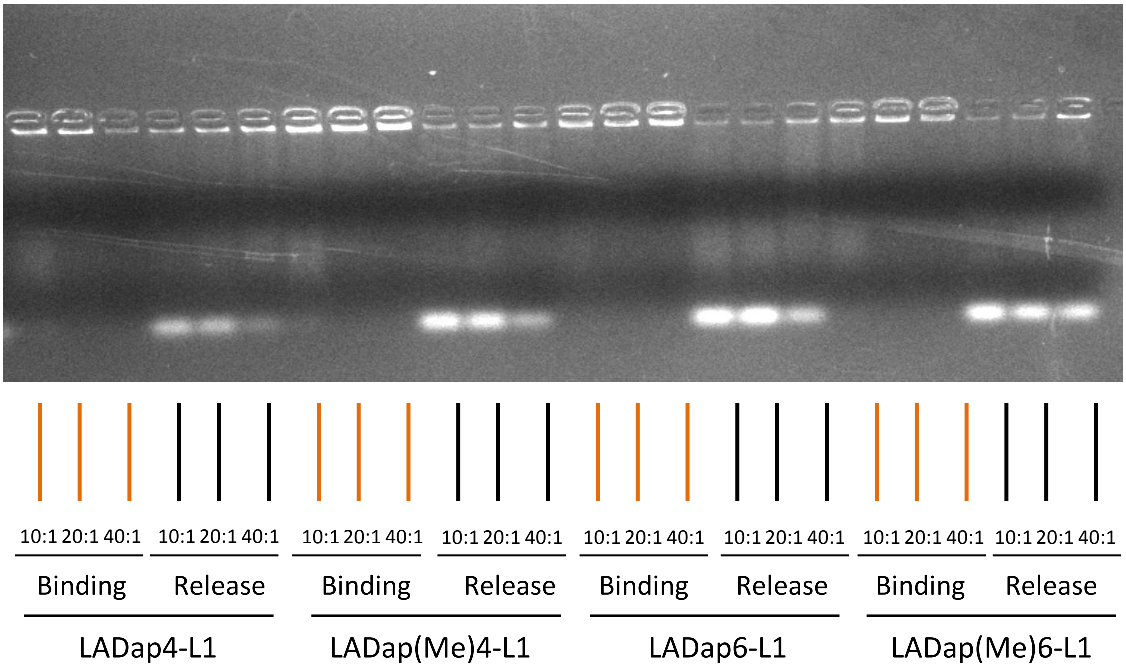

Supplementary Figure 14. *The effects of BALF on parameters related to siRNA delivery. The binding and disassociation of siRNA from various peptides in the presence of 50% BALF (**B**). Peptide to siRNA mass ratios are given and agarose gels run north to south show binding (no migration shows complete binding) and release with 50% BALF. Release is incomplete for LADap4-L1.*
