# Supplementary material for: Manipulating the pH response of 2,3-diaminopropionic acid rich peptides to mediate highly effective gene silencing with low-toxicity
Source: J Control Release. 2013 Dec 28;172(3):929–38. doi: 10.1016/j.jconrel.2013.09.033 (PMC3858832; doi:10.1016/j.jconrel.2013.09.033)
Supplement: Supplementary file 2 — Supplementary material and methods. [file mmc2.pdf]

SUPPLEMENTAL MATERIAL AND METHODS

Circular dichroism experiments - Peptides were dissolved in 5 mM Tris amine without pH adjustment at a final concentration of between 30 and 50 μM. The samples were titrated down by adding 0.3% or 1% (v/v) $HClO_4$ solution in micro-litre amounts. ClO_4- is optically transparent hence the substitution of HCl (which has substantial absorbance below 200 nm) by $HClO_4$ makes a significant improvement on spectral quality in the far-UV region. CD spectra were acquired on a Chirascan™ Spectrometer (Applied Photophysics, Leatherhead, UK) with samples maintained at 310 K. For pH titration experiments, far-UV CD spectra were obtained with the samples titrated down by adding 0.3% or 1% (v/v) $HClO_4$ solution in micro-litre amounts. Spectra were recorded from 260 to 180 nm using a 0.5 mm path length and were processed using Chirascan software where a spectrum of the peptide free solution was subtracted and Savitzky-Gorlay smoothing with a convolution width of 5 points applied. Results for all CD assay measurements are expressed as an average of two or more independent repeated experiments.

Sample preparation for solid-state NMR - The lipids 1-palmitoyl-2-oleoyl-sn-glycero-3-phosphatidylcholine (POPC), 1-palmitoyl$_{d31}$-2-oleoyl-sn-glycero-3-phosphatidylcholine (POPC-d31), 1-palmitoyl$_{d31}$-2-oleoyl-sn-glycero-3-phosphatidylserine (POPS-d31) and cholesterol were obtained from Avanti Polar Lipids, Inc. (Alabaster, AL) and used without further purification. All other reagents were analytical grade or better. Samples with the lipid composition POPC/POPC-d31/cholesterol (85:15:30) or POPC/POPS-d31/cholesterol (85:15:30) were prepared. A total of around 5 mg lipids per sample were dissolved and mixed in chloroform and dried under rotary-evaporation at room temperature. In order to remove all organic solvent, the lipid films were exposed to vacuum overnight. The films were then rehydrated with 4 ml of a suspension of LADap(Me)4-L1 or LADap(Me)6-L1 in 10 mM Tris/piperazine buffer at various pH at room temperature. For wideline ^{2}H NMR experiments peptides were added to the lipids at 2% by mol. Samples were subjected to five rapid freeze-thaw cycles for further sample homogenization, generating multi-lamellar vesicles, and then centrifuged at 21000 g for 30 min at room temperature. The pellets, containing lipid vesicles and associated peptides were transferred to Bruker 4 mm MAS rotors for NMR measurements. Lipid vesicles were also prepared in this way in the absence of peptide.

Solid-state NMR - ^{2}H quadrupole echo experiments [39] for samples containing either POPC-d31 or POPS-d31 were performed at 61.46 MHz on a Bruker Avance 400 NMR

spectrometer using a 4 mm MAS probe, spectral width of 100 KHz and with recycle delay, echo delay, acquisition time and 90º pulse lengths of 0.25 s, 100 μs, 2.6 ms and 3 μs respectively. The temperature was maintained at 310 K to keep the bilayers in their liquid-crystalline phase. During processing the first 10 points were removed in order to start Fourier-transformation at the beginning of the echo. Spectra were zero filled to 1k points and 50 Hz exponential line-broadening was applied. Smoothed deuterium order parameter profiles were obtained from symmetrised and dePaked ^{2}H-NMR powder spectra of either POPC-d31 or POPS-d31 according to published procedures [40-42] using the Amix software (Bruker, Karlsruhe, Germany). Order parameters were averaged along the length of the acyl chain and the difference in average order between peptide free and peptide containing liposomes was used to calculate a pK_{mem} for the peptide induced disordering of the deuterated reporter lipids.

Further Materials - Dulbecco's modified eagle medium (DMEM), RPMI-1640 medium, Opti-MEM 1 reduced serum medium, antibiotic-antimycotic liquid, fetal bovine serum (FBS) and Lipofectamine 2000™ were purchased from Invitrogen (CA, USA). The luciferase assay system was purchased from Promega (Madison, WI, USA). GelRed™ nucleic acid stain was purchased from Biotium (Hayward, CA, USA). GAPDH primary antibody was purchased from Abcam (MA, USA). Amersham ECL™ Western Blotting detection reagents were purchased from GE Healthcare (Amersham, UK). Plasmid DNA (gWIZ™ Luciferase) was purchased from Aldevron (Fargo, ND, USA). siRNAs (Silencer®/Silencer® Select GAPDH positive control siRNA and Silencer®/Silencer® Select negative control siRNA) and siPORT™ transfection agent were purchased from Ambion (Austin, TX, USA) and have been extensively tested by the manufacturers, showing minimal off-target effects.

Cell culture and DNA transfection - A549, MCF-7 and THP-1 cells were obtained from ATCC (Manassas, VA, USA). The cells were maintained at 5% CO2, 37°C in DMEM supplemented with 10% FBS, 100 units/ml penicillin, 100 μg/ml streptomycin and 0.25 μg/ml amphotericin B. The cells were subcultured once weekly. Cells were transfected with peptide/DNA complexes at 1 μg luciferase /well in 24-well plates and with peptide to DNA weight ratios of 5:1, 10:1, 15:1 and 20:1 with 10:1 generally giving the best results. The cells seeding density was 5 × 104 cells per well. The complexes were prepared in Opti-MEM I reduced serum medium. After 4 hours of incubation at 37°C, the cells were washed with PBS and fresh DMEM supplemented with 10% FBS were added to the cells. After 48 hours, the luciferase expression was detected using the luciferase assay system according to the

manufacturer's protocol. The cells were transfected with Lipofectamine 2000™ at 2 μl per well under the same conditions.

siRNA transfection - A549, MCF-7 and THP-1 cells, were transfected with peptide/siRNA complexes containing 50 or 100 pmol GAPDH siRNA or negative control siRNA/well in 6-well plates, giving a final siRNA concentration of 50 or 100 nM. Cells were seeded at the following densities; A549 and MCF-7 - 1×10^5 cells per well, THP-1 in suspension – 5×10^5 cells per well, differentiated THP-1 – 1×10^6 cells per well. Peptides were added at peptide/siRNA w/w ratios of 5:1, 10:1 and 15:1 with 10:1 ratios generally giving the best results. The complexes were prepared in Opti-MEM I reduced serum medium. After 4 hours of incubation at 37°C, the cells were washed with PBS and fresh DMEM supplemented with 10% FCS were added to the cells. After 72 hours, the cells were washed with cold PBS and lysed. The cell extracts containing 20 μg of protein were loaded into a 10% SDS-polyacrylamide gel and electrophoresis was run at 150 V for 60 minutes. After the proteins were resolved, they were transferred into a nitrocellulose membrane which was blocked in 5% non-fat dry milk for 2 hours with shaking. The membrane was washed and incubated with primary antibody overnight at 4°C on shaking. After rinsing the membrane was incubated with horseradish peroxide conjugated secondary antibody for 2 hours at room temperature. The bound secondary antibodies were detected with ECL™ Western blotting detection reagents. For all Western Blot experiments, densitometry was performed using Image J software (version 1.46) and is reported as an average of three independent repeats. The cells were transfected with Lipofectamine 2000™ or siPORT™ at 2.5 μl (50 pmol siRNA) and 5 μl (100 pmol siRNA) per well under the same conditions.

Cytotoxicity assay - For undifferentiated THP-1 cells, cell suspensions were seeded on 24 well plates at a density of 1 x 10^5 cells in 500 μl media per well. For differentiated THP-1 cells, the cells were seeded at a density of 2 x 10^5 in 200 μl per well and were differentiated with 100 ng/ml PMA. After 48 hours, the cells were fasted overnight. Various amount of peptides, Lipofectamine 2000™, siPORT™ and siRNA complexes were added to the cells in Opti-MEM I reduced serum medium. Peptides were added at a 10:1 peptide/siRNA w/w ratio with the benchmark compounds added at the same levels required for optimum transfection. 20 pmol siRNA was added to each well giving final siRNA concentrations of 40 and 100 nM for undifferentiated monocyte and differentiated macrophage THP-1 cells respectively.

HUVEC cells were maintained in F-12K media supplemented with 10% FBS, 1% antibiotics, 15 IU/ml heparin and 30 μg/ml endothelial cell growth supplement (ECGS). HUVEC cells were plated in a 96-well plate at the cell density of 2 x 10^4 cells/well. The peptide/siRNA or Lipofectamine 2000/siRNA complexes (15 pmol siRNA, peptides to siRNA 10:1 w/w ratio, 0.7 μl Lipofectamine 2000™ per well) were prepared in serum free OptiMEM.

After 4 hours of incubation at 37°C, the cells were washed with PBS and replaced with serum supplemented RPMI-1640 medium. At 24 hours post-transfection, cells were washed with PBS again. MTT solution (0.8 mg/ml) was added to cells which were incubated at 37°C for 4 hours. The insoluble formazan was dissolved in isopropanol for 30 min at room temperature on shaking. Cell viability was assessed by measuring the absorbance at 570 nm. The results were expressed as percentage of the A570nm for cells treated with peptides against that obtained for untreated cells and are an average of three independently repeated duplicate experiments.

Confocal live cell imaging - Both differentiated and suspension THP-1 cells, A549 cells and HUVECs were seeded on a 35 mm Mattek glass bottom culture dish (Mattek Corp. Ashland, MA) with 1 × 10^5 cells per well and incubated overnight. The cells were further incubated for 20 hours before imaging. LAH4-L1/siRNA or LADap(Me)6-L1/siRNA complexes containing 2 μg of siGLO (Cy3-siRNA) prepared in 1 ml of Opti-MEM I were added to the cells which were incubated for 30 minutes. The cells were then washed with warm PBS twice and replaced with fresh serum supplemented DMEM. The cells were imaged at 4 hours and 24 hours post-transfection. At 5 minutes prior to imaging, the medium was replaced with Opti-MEM I. For lysosomes labelling, Lysotracker Green DND-26 (50 nM) was added to the cells 2 minutes before imaging. During live cell imaging, the cells were incubated at 37°C and 5% CO_2 in a heated Perspex box. For confocal imaging involving inhibitors, the experiments were carried out as described above except the cells were treated with nystatin (25 μg/ml) or chlorpromazine (10 μg/ml) for 30 minutes before the addition of and during the incubation with the complexes. Confocal laser scanning microscopy (Carl Zeiss LSM 510 Meta/Axicam system, Germany) with argon laser (488 nm) and HeNe laser (543 nm) was used to visualize the cells. The microscope was equipped with Plan-Apochromat 64x/1.4 oil DIC objective lens. The images were analysed with Zeiss LSM Image Browser (Version 4.2.0.121).

Particle Size Measurement - Peptide/DNA or peptide/siRNA complexes were prepared at various weight ratios. Nucleic acids solution and peptide solution were prepared separately in 150 mM NaCl. Equal volumes of nucleic acids and peptide solution were mixed together to give a final volume of 50 µl containing 2 µg of nucleic acids. After leaving for 30 minutes at room temperature, the hydrodynamic diameter of the complexes was measured by Photon Correlation Spectroscopy (Delsa™Nano C, Beckman Coulter, CA, USA).

Gel retardation assay – Peptide/DNA or peptide/siRNA complexes were prepared at various ratios with 0.2 µg siRNA in 10 µl TAE buffer pH 7.4. After leaving the complexes for 30 minutes at room temperature, the samples were loaded into either 1% or 2% w/v agarose gel, for DNA and siRNA respectively, containing GelRed™ nucleic acids stain. To assess release properties, 5 µl of 2 mM SDS was added to samples to release the DNA/siRNA. Gel electrophoresis bound and released samples were run in TAE buffer at 100 V for either 60 or 20 minutes, for DNA and siRNA respectively, and the gel was visualized under the UV illumination. For the nuclease protection assay, the complexes were prepared as described above. After 30 minutes, the complexes were incubated with 80 ng of RNase A for 30 minutes at 37°C to allow degradation of unprotected siRNA. The activity of RNase was then stopped by the addition of 1 µl of RNase inhibitor. 5 µl of 2 mM SDS was added to the samples to release the protected siRNA. The resulting mixtures were incubated at room temperature for 1 hour. The samples were then loaded into a 2% w/v agarose gel and electrophoresis was run as described above. Untreated siRNA, SDS treated siRNA and RNase treated siRNA were used as controls.

Flow cytometry study of the intracellular uptake of peptide/siRNA complexes - A549 cells were seeded in 6-well plate at a density of 2×10^5 cells per well and were incubated for 24 hours. LAH4-L1/siRNA or LADap(Me)6-L1/siRNA complexes containing 2 µg of siGLO (Cy3-siRNA) prepared in 1 ml of Opti-MEM I or 50% BALF (v/v) were added to the cells which were incubated for 2 hours. Cells treated with either Opti-MEM I or 50% BALF without peptide/siGLO (Cy3-siRNA) complexes served as control. The cells were then washed with warm PBS twice, trypsinized and collected. To quench extracellular fluorescence signal, trypan blue solution in PBS (0.04%) was added to the cells for 2 minutes. The cells were spun down and washed with cold PBS solution thrice. Finally, the cells were re-suspended in 1 ml cold PBS solution and sieved with a cell strainer (BD Bioscience, USA). Ten thousand cells were monitored and evaluated by FACSCantoII Analyzer (BD Bioscience,

USA) to determine the cellular uptake of peptide/cy3-siRNA complexes. Cellular uptake (%) was defined as the percentage of cells with positive fluorescence signal compared to control.

The effects of BALF on parameters related to siRNA delivery - Peptide/siRNA complexes were prepared at various ratios with 0.2 µg siRNA in 10 µl TAE buffer, pH 7.4. After leaving the complexes for 30 minutes at room temperature, the samples were loaded into 2% w/v agarose gel, containing GelRed™ nucleic acids stain. To assess the stability of complexes in BALF, 10 µl of BALF was added to samples and incubated at 37 °C for 30 minutes. Gel electrophoresis was run in TAE buffer at 100 V for 20 minutes and the gel was visualized under the UV illumination.

Statistical analysis - Statistical analysis was by ANOVA with Bonferonni post-hoc t-test, one tailed.

SUPPLEMENTARY REFERENCES

[39] Davis, J.H. (1983) The description of membrane lipid conformation order and dynamics by 2H-NMR, Biochim. Biophys. Acta 737, 117-171.

[40] Schäfer, H., Mädler, B. and Volke, F. (1995) De-PAKE-ing of NMR powder spectra by nonnegative least- squares analysis with Tikhonov regularization. J. Magn. Reson. 116, 145-149.

[41] Sternin, E., Bloom, M., and MacKay, A.L. (1983) De-PAKE-ing of NMR Spectra. J. Magn. Reson. 55, 274-282.

[42] Seelig, A. and Seelig, J. (1974) Dynamic structure of fatty acyl chains in a phospholipid bilayer measured by deuterium magnetic-resonance. Biochemistry 13, 4839-4845.
